# Cellular automaton models for time-correlated random walks: derivation and analysis

**DOI:** 10.1038/s41598-017-17317-x

**Published:** 2017-12-05

**Authors:** J. M. Nava-Sedeño, H. Hatzikirou, R. Klages, A. Deutsch

**Affiliations:** 1 Technische Universität Dresden, Center for Information Services and High Performance Computing, Nöthnitzer Straße 46, 01062 Dresden, Germany; 2 Department of Systems Immunology and Braunschweig Integrated Centre of Systems Biology, Helmholtz Center for Infection Research, Inhoffenstraße 7, 38124 Braunschweig, Germany; 3 School of Mathematical Sciences, Queen Mary University of London, Mile End Road, London, E1 4NS United Kingdom

## Abstract

Many diffusion processes in nature and society were found to be anomalous, in the sense of being fundamentally different from conventional Brownian motion. An important example is the migration of biological cells, which exhibits non-trivial temporal decay of velocity autocorrelation functions. This means that the corresponding dynamics is characterized by memory effects that slowly decay in time. Motivated by this we construct non-Markovian lattice-gas cellular automata models for moving agents with memory. For this purpose the reorientation probabilities are derived from velocity autocorrelation functions that are given a priori; in that respect our approach is “data-driven”. Particular examples we consider are velocity correlations that decay exponentially or as power laws, where the latter functions generate anomalous diffusion. The computational efficiency of cellular automata combined with our analytical results paves the way to explore the relevance of memory and anomalous diffusion for the dynamics of interacting cell populations, like confluent cell monolayers and cell clustering.

## Introduction

Within the past two decades transport processes in many branches of the sciences were observed to be *anomalous*, in the sense that they do not obey the laws of conventional statistical physics and thermodynamics^1–8^. Important cases are diffusion processes where the long-time mean square displacement (MSD) does not grow linearly in time. That is, $\langle {r}^{2}\rangle \propto {t}^{\varphi }$, where the angular brackets denote an ensemble average, does not increase with *ϕ* = 1 as expected for Brownian motion but either *subdiffusively* with *ϕ* < 1 or *superdiffusively* with *ϕ* > 1^9–11^. After pioneering work on amorphous semiconductors^12^, more recently anomalous diffusion has been detected in many other complex systems^3,4,6,8^; here well-known examples of physcial systems are nanopores^13^, plasmas^14^ and glassy material^15^.

Biological systems frequently exhibit anomalous properties as well: Prominent examples are the foraging of organisms^16^, epidemic spreading^17^ and the diffusion of macromolecules in biological cells^6^. Especially, it was found that many types of cells migrate anomalously: *Hydra* cells^18^, mammary gland epithelial cells^19^, *MDCKF* cells^20^, amoeboid *Dictyostelium* cells^21,22^, T cells^23^, breast carcinoma cells^24^ and stem cells^25^ were all experimentally observed to move superdiffusively, typically with non-Gaussian position and/or velocity distribution functions^18–24^ accompanied by either exponential or non-exponential position^23^, and exponential^21,22,26^ or power law^18–20^ velocity autocorrelation function (VACF) decay. For T cells it was argued that superdiffusion optimizes their search to kill intruding pathogens^23,27^. While all these results are on single cell migration, currently collective cell migration is moving into the center of interest^28^, where cells interact with each other, e.g., by chemical signalling^29^. Interesting phase transitions inside dense tissues of epithelial cell monolayers were reported^30^ and partially traced back to particular features of single-cell migration^31^. It was also observed experimentally that superdiffusion appears to foster the formation of clusters of stem cells leading to tissue formation^25^. Other works investigate the role of interacting agents for phase transitions in active matter^32^, and collective anomalous dynamics emerging from the interaction of single agents^33,34^.

On the theoretical side there are many different ways to model anomalous diffusion in terms of stochastic processes, such as continuous time random walks (CTRW)^1,5^, generalized Langevin equations^2^, Lévy flights and walks^8^, fractional diffusion equations^1,4,5^, scaled Brownian motion and heterogeneous diffusion processes^7^. A subset of these models, most notably generalized Fokker-Planck equations^18–20^ generalized Langevin equations^21,22^, and generalized random walks^23,24^, has been used to model anomalous movement of single biological cells. However, solving equations for anomalously moving single particles analytically or numerically is typically difficult already^1,2,4,5,7,8^. To our knowledge there exists no systematic attempt to generalize this theory to model interacting many-particle systems; the only exception we are aware of is a line of work in plasma physics^14^.

On the other hand, several models have been introduced to study the collective movement of particles and cells^35–37^. Cellular automata (CA) in particular have the advantage of being less computationally demanding than continuous models when performing simulations. A specific type of CA is the so-called lattice-gas cellular automata (LGCA)^38^. In LGCA, each lattice node can contain several particles, which at each time step are rearranged within the lattice node according to the interaction rule, and subsequently moved to a neighboring node. In a biological context particles can be regarded as cells, while the LGCA rules mimic cell migration and interaction. Furthermore, LGCA have proved to be amenable to mathematical analysis^39^. For this reason, LGCA have been introduced as mesoscopic models for single and collective cell migration^40–43^. So far, none of the mentioned models has considered anomalous migration of single cells.

It thus arises the need to design simple fundamental schemes by which the collective properties of interacting agents can be studied whose individual dynamics is anomalous. Using our methods will enable to explore the relevance of microscopic single-particle dynamics for emerging collective phenomena. We thus devise a scheme by which anomalous dynamics of many interacting agents can be simulated efficiently, which is based on capturing the non-trivial decay of VACFs. This approach generates superdiffusion if the correlation decay is of power law-type^18–20^. We emphasize that our data-driven approach can be applied to any moving entity that exhibits dynamics with non-trivial correlation decay, a feature that may be expected to hold more generally for the movement of biological organisms^16^.

**Figure 1:**
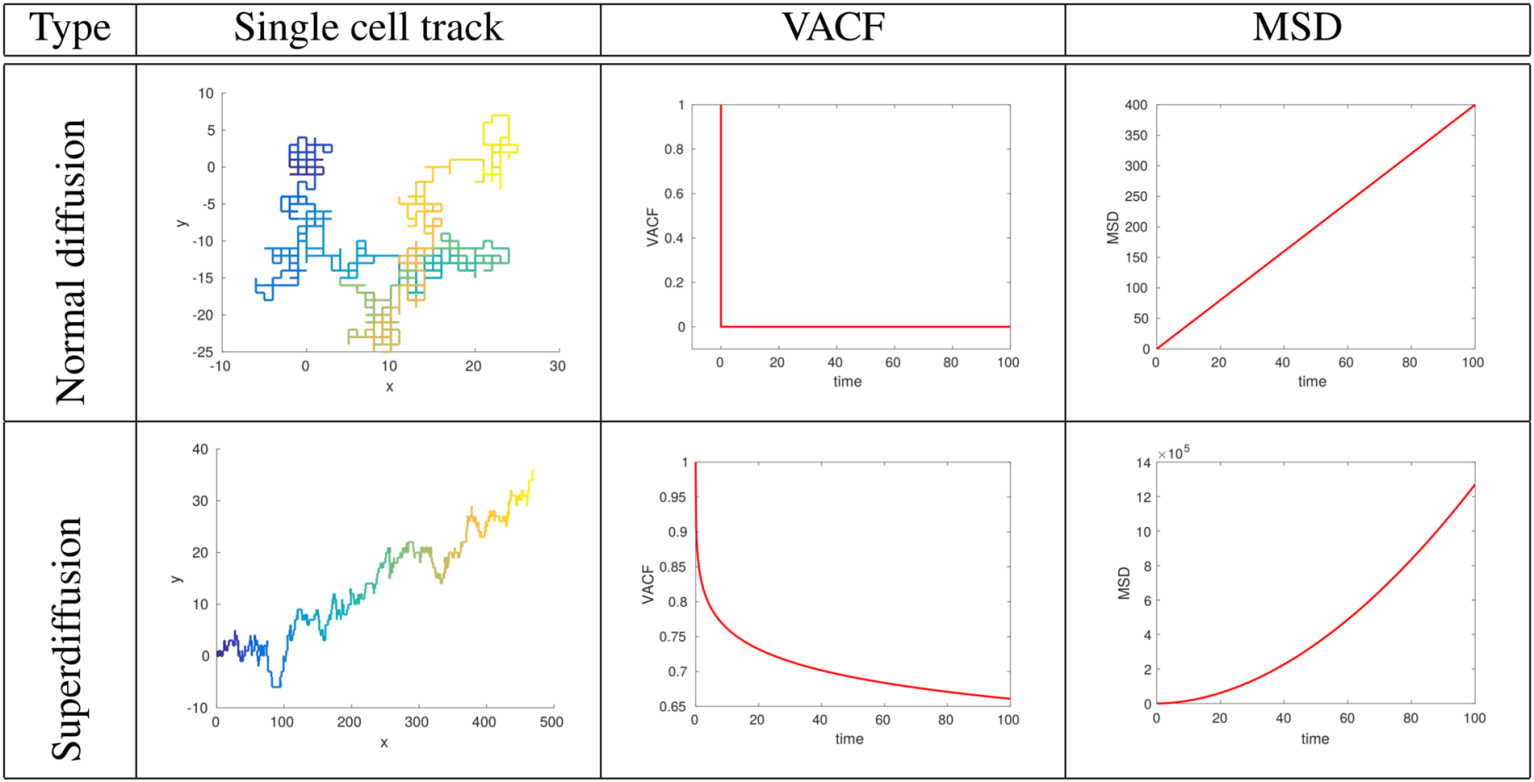
Basic types of diffusive movement in two dimensions characterized by two key quantities (VACF and MSD). Shown in the first column are the tracks of a single particle starting at (*x*, *y*) = (0, 0) that exhibits either long-time normal diffusion (top row) or superdiffusion (bottom row). The color gradient changes from blue to yellow with elapsed time. The second column displays the particle’s velocity autocorrelation function (VACF) Eq. (3), the third column its mean square displacement (MSD) Eq. (4).

We use the LGCA modeling framework and construct various time-correlated random walk models. After briefly introducing the LGCA concept, we define an LGCA model for unbiased random walk. Next, motivated by the biophysical mechanism of single cell crawling we construct a *persistent random walk* LGCA model wherein angular (orientation) correlations give rise to temporal correlations. Subsequently, we construct a first LGCA model for *time-correlated random walk* which is data-driven, as the model’s reorientation probabilities are derived by assuming that the exact temporal dependence of the VACF is known a priori. Finally, we develop a *generalized time-correlated random walk* LGCA model for cell movement at short and medium time regimes by curing a deficiency of our first time-correlated random walk model for short times. Figure 1 shows single cell tracks with the corresponding VACFs and MSDs for our main two classes of LGCA models we are dealing with, which are Markovian and non-Markovian random walks, exemplified by showing their basic features.

## Lattice-gas cellular automata

**Figure 2:**
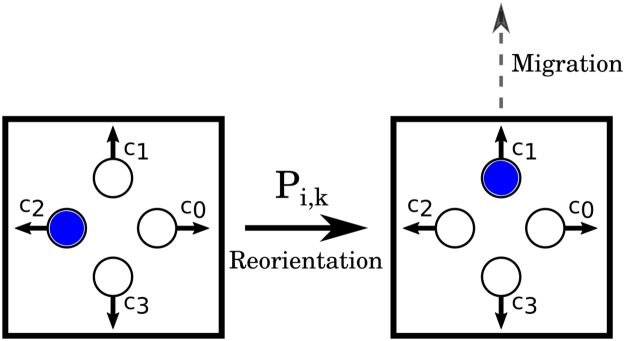
LGCA dynamics. At each time step a particle is assigned an orientation ${\overrightarrow{c}}_{i}$ with a probability *P*_*i,k*_. Subsequently, the particle is translocated to the nearest neighbor in the direction of its orientation.

Cellular automata are mathematical models where the states of discrete lattice nodes are updated at discrete time steps. If the states of the lattice sites are Boolean, such states can be interpreted as presence/abscence of a particle at a particular node. The lattice-gas cellular automaton is a specific CA type, which has two important characteristics: first, particle reorientation and migration are separated into a probabilistic and a deterministic step, respectively. Secondly, to each node, *b* velocity channels are associated which can be occupied by at most one particle (exclusion principle). The set of velocity channels is given by ${\overrightarrow{c}}_{j}=(\cos \,\frac{2\pi j}{b},\,\sin \,\frac{2\pi j}{b})$, j∈ {0, 1, …, *b* − 1} (see Fig. 2). Particles move in discrete time steps of duration *τ* to neighboring nodes located a distance *ε* away in the lattice. At each time step particles adopt the orientation ${\overrightarrow{c}}_{i}$ with a probability *P*_*i,k*_, called the reorientation probability, where *t* = *kτ*, $k\in {\mathbb{N}}$ is the elapsed simulation time. Subsequently, cells will be deterministically translocated to the nearest neighbor located in the direction of ${\overrightarrow{c}}_{i}$; see Fig. 2 for a sketch of LGCA dynamics.

If we were to have *N* different particles in a single node, then the probability of the particles adopting the orientations ${\overrightarrow{c}}_{{n}^{1}},{\overrightarrow{c}}_{{n}^{2}},\ldots ,{\overrightarrow{c}}_{{n}^{N}}$ would be given by

1 $${P}_{{n}^{1},\ldots ,{n}^{N},k}=\prod _{\ell =1}^{N}{P}_{{n}^{\ell },k}.$$

## Classical random walk

Here we define an LGCA model for unbiased random walk^44^. In this model, at all timesteps, all orientations are chosen with equal probability^45,46^. This means that the reorientation probability is given by

2 $${P}_{i,k}=\frac{1}{b}.$$

In order to characterize the movement of a particle in this model we calculate time-dependent expressions for the VACF and the MSD, which measure the persistence, in terms of memory decay in time, and the spatial exploratory power of a moving particle, respectively. In LGCA models space, time and particle velocities are discrete so that the VACF is given by^47^

3 $$g(k)=\langle {\overrightarrow{c}}_{{i}_{0}}\cdot {\overrightarrow{c}}_{{i}_{k}}\rangle =\sum _{{i}_{k}=1}^{b}{P}_{{i}_{k},k}[{\overrightarrow{c}}_{{i}_{0}}\cdot {\overrightarrow{c}}_{{i}_{k}}],$$

where ${\overrightarrow{c}}_{{i}_{k}}$ is the orientation of the particle at the *k*-th time step. The MSD is calculated by

4 $$\langle {r}_{k}^{2}\rangle =\sum _{{r}_{k}}{r}_{k}^{2}{P}_{{r}_{k},k},$$

where *r*_*k*_ is the norm of the particle displacement at time step *k* defined as ${\overrightarrow{r}}_{k}={\overrightarrow{x}}_{k}-{\overrightarrow{x}}_{0}$, where ${\overrightarrow{x}}_{k}$ is the position of the particle at time step *k*. The probability ${P}_{{r}_{k},k}$ can be calculated from Eq. (2) by noticing that ${\overrightarrow{r}}_{k}=\varepsilon {\sum }_{k}{\overrightarrow{c}}_{{i}_{k}}$. In this simple random walk model Eq. (3) reduces to a sum of cosines over homogeneously distributed angles. Hence the VACF is given by *g*(*k*) = *δ*_0,*k*_, where *δ*_*i,j*_ is the Kronecker delta. In the limit *τ* → 0 the VACF tends to

5 $$g(t)=\delta (t),$$

where *δ*(*t*) is the Dirac delta function. Equation (5) means that the movement of the particle is uncorrelated as soon as it starts moving, i.e. the orientation of the particle at any time step *k* is completely independent from its previous orientation, which is the Markov property. On the other hand, simple combinatorics can be used to calculate the particle’s MSD yielding $\langle {r}_{k}^{2}\rangle =k{\varepsilon }^{2}$. We can rewrite this expression by using the general definition of the diffusion coefficient

6 $$D=\mathop{\mathrm{lim}}\limits_{t\to \infty }\frac{\langle {r}_{t}^{2}\rangle }{2dt},$$

where *d* is the dimension of space. For a memoryless random walk this equation boils down to ${D}_{rw}=\frac{{\varepsilon }^{2}}{2d\tau }$ with an MSD of $\langle {r}_{k}^{2}\rangle =2d{D}_{rw}k\tau$. Given that *τ* is the time step length and that *t* = *kτ* is the elapsed time, the MSD is

7 $$\langle {r}_{t}^{2}\rangle =2d{D}_{rw}t.$$

Equation (7) shows that the classical random walk model trivially yields a normal diffusion process, where the MSD increases linearly in time^48^.

## Persistent random walk

The assumption of the classical random walk in Eq. (2) that all the directions of movement are equally probable is generally not true. We will now construct an LGCA model motivated by a simple biophysical model for a single, persistently moving cell.

### Rule derivation

**Figure 3:**
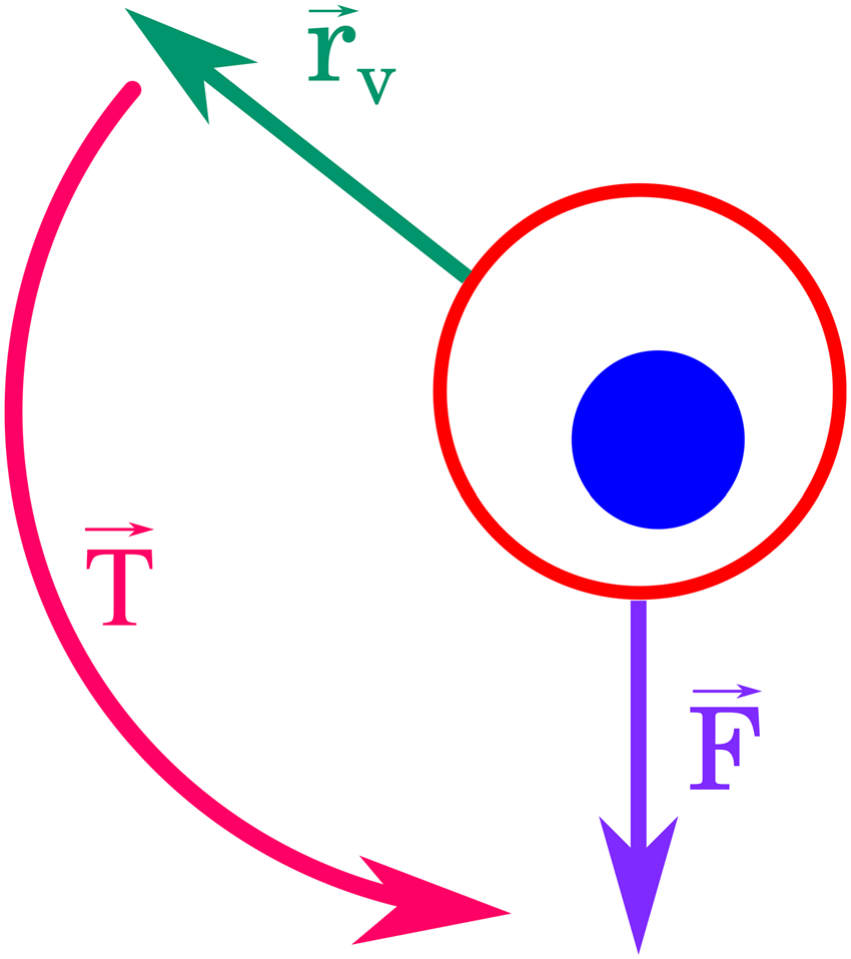
Reorientation of a biological cell moving persistently. A cell which moves in a direction ${\overrightarrow{r}}_{v}$ feels an intracellular force $\overrightarrow{F}$ and reorients towards the direction of the force due to the torque $\overrightarrow{T}$.

Biological cells move by exerting forces to propel themselves. In the case of eukaryotic cells such as fibroblasts, movement is achieved by crawling over the substrate. Crawling is performed by polymerization of the actin cytoskeleton at the leading edge propelling the cell in this direction. We can identify the direction of the actin concentration gradient with the direction of movement of a cell ${\overrightarrow{r}}_{v}$. Furthermore, intracellular forces due to actin activity $\overrightarrow{F}$ point towards the direction of new actin polymerization. The cell then rotates/reorients/repolarizes due to the torque defined by $\overrightarrow{T}={\overrightarrow{r}}_{v}\times \overrightarrow{F}$, whose norm is given by

8 $$T={r}_{v}F\,\sin ({\theta }_{F}-{\theta }_{r}),$$

where $T=\parallel \overrightarrow{T}\parallel$, ${r}_{v}=\parallel {\overrightarrow{r}}_{v}\parallel$, $F=\parallel \overrightarrow{F}\parallel$, and *θ*_*r*_ and *θ*_*F*_ are the angular components of ${\overrightarrow{r}}_{v}$ and $\overrightarrow{F}$, respectively; see Fig. 3. In the overdamped regime, characteristic of the cellular environment, the intracellular force $\overrightarrow{F}$ will not cause the cell to rotate indefinitely but rather will cause the cell to rotate until ${\overrightarrow{r}}_{v}$ and $\overrightarrow{F}$ are parallel. Taking this into account, it is possible to rewrite Eq. (8) as $T={r}_{v}F\,\sin ({\theta }_{rt}-{\theta }_{r})$, where *θ*_*rt*_ is the direction of motion of the cell after it has finished its reorientation.

The torque is given in terms of an energy of rotation *U*(*θ*_*r*_, *θ*_*rt*_) as $\overrightarrow{T}=-\frac{\partial U({\theta }_{r},{\theta }_{rt})}{\partial {\theta }_{r}}$. Using this equation, the energy of rotation is then given by

9 $$U({\theta }_{r},{\theta }_{rt})=-\upsilon \cos ({\theta }_{rt}-{\theta }_{r}),$$

where *υ* = *r*_*v*_ *F* is the amplitude of the torque generated inside the cell. Having defined the energy of rotation, Eq. (9), we can describe the cell’s reorientation by a Langevin equation^49^ as $\frac{\partial {\theta }_{r}}{\partial t}=-\gamma \frac{U({\theta }_{r},{\theta }_{rt})}{\partial {\theta }_{r}}+\xi (t)$ with relaxation constant *γ* and a zero-mean, delta correlated noise term *ξ*(*t*) such that $\langle \xi (t)\xi (t^{\prime} )\rangle =2{D}_{\theta }\delta (t-t^{\prime} )$, where *D*_*θ*_ is the rotational diffusion coefficient. Based on this we can immediately derive the LGCA reorientation probabilities^50^.

These probabilities for a single cell then read

10 $${P}_{{i}_{k},k}=\frac{1}{Z}\exp [\beta ({\overrightarrow{c}}_{{i}_{k-1}}\cdot {\overrightarrow{c}}_{{i}_{k}})],$$

where ${\overrightarrow{c}}_{{i}_{k-1}}$ is the orientation of the cell at the previous time step, *Z* is the normalization constant (also known as the partition function) and $\beta =\frac{\tilde{\gamma }}{{D}_{\theta }}$ is the sensitivity, where $\tilde{\gamma }=\upsilon \gamma$ is the effective relaxation constant.

### Model analysis and results

Using Eq. (10) we can calculate the VACF and MSD for this model. By using the properties of the partition function *Z* in Eq. (10) the VACF at every time step *k* is (see Sec. B in the Supplementary Information)

11 $$g(k)=\exp (\alpha k),$$

where the exponent *α* depends heavily on the lattice dimension and geometry. In particular, in a 2D square lattice we have $\alpha =\,\mathrm{ln}[\tanh (\frac{\beta }{2})]$. In all geometries the exponent is *α* < 0 over its domain *β* > 0 (see again Sec. B in the Supplementary Information).

Equation (11) can be generalized to continuous time and space by employing the relations between the time and space scalings, namely the diffusion coefficient in the random walk limit *D*_*rw*_ and the instantaneous cell speed $v=\frac{\varepsilon }{\tau }$, where *ε* is the lattice spacing and *τ* the time step length. Taking the limit *τ* → 0 yields the VACF in continuous time and space

12 $$g(t)=\exp (\frac{\alpha {v}^{2}}{2d{D}_{rw}}t).$$

When time is discrete the MSD is given by^51,52^ (see Sec. A in the Supplementary Information) $\langle {r}^{2}\rangle =2d{D}_{rw}k\tau +$ $\langle {\sum }_{i\mathrm{=1}}^{k}{\sum }_{j\mathrm{=1}}^{k}{v}^{2}{\tau }^{2}\,\cos ({\theta }_{i}-{\theta }_{j})(1-{\delta }_{ij})\rangle$. Calculating the expected value on the right hand side we obtain (see again Sec. A in the Supplementary Information)

13 $$\langle {r}_{k}^{2}\rangle =2d{D}_{rw}k\tau +2{v}^{2}\sum _{i=1}^{k}(k\tau -i\tau )g(i)\tau .$$

Using Eq. (11) and taking the limit of small time step length we get

14 $$\langle {r}_{t}^{2}\rangle =2d{D}_{rw}t+2{v}^{2}{\int }_{0}^{t}(t-\tau ){e}^{\frac{{v}^{2}\alpha }{2d{D}_{rw}}\tau }{\rm{d}}\tau ,$$

which can be easily integrated to obtain the MSD for continuous time

15 $$\langle {r}_{t}^{2}\rangle =2d{D}_{rw}t(1-\frac{2}{\alpha })+{(\frac{2\sqrt{2}d{D}_{rw}}{v\alpha })}^{2}[\exp (\frac{{v}^{2}\alpha t}{2d{D}_{rw}})-1].$$

Equation (14) agrees with the formal solution of the MSD for an overdamped Langevin equation with colored noise^53^. Correspondingly, Eq. (10) coincides with a Langevin process where the noise is not white but colored whose correlation is given by Eq. (12). Using Eq. (15) we find that $\langle {r}^{2}\rangle \propto t$ when *t* → ∞ rather quickly.

**Figure 4:**
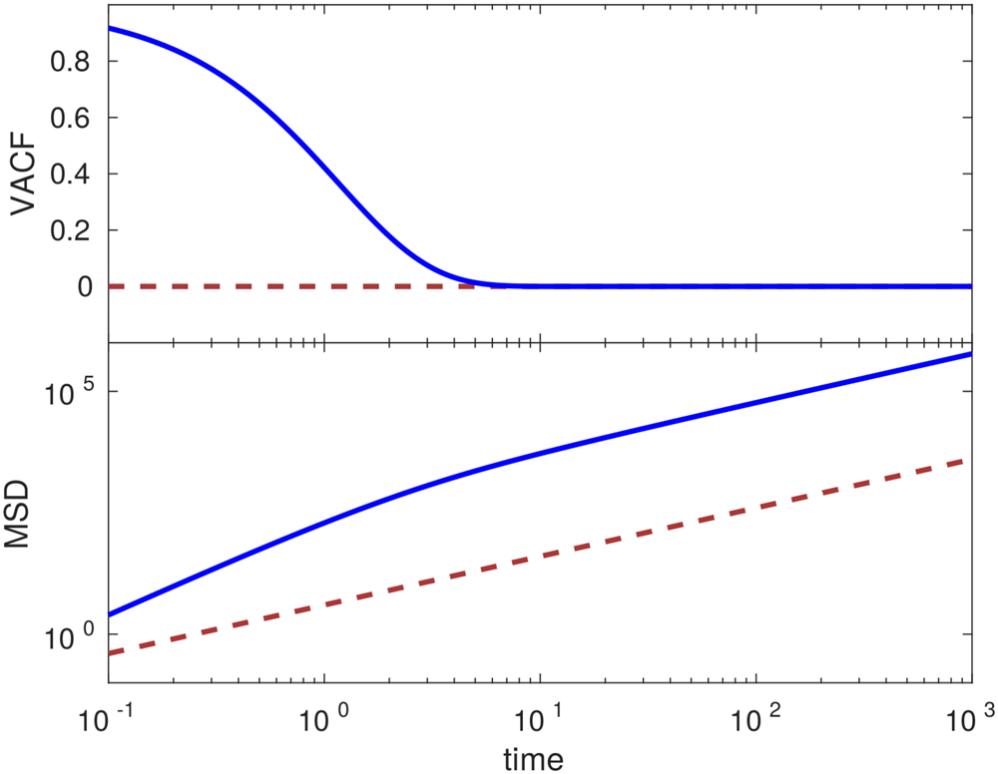
Comparison between random walk and persistent random walk models. Shown are theoretical results for VACF (top) and MSD (bottom) in the random walk (dashed maroon line) and persistent (solid blue line) models. The parameter values are D_*rw*_ = 1, *v* = 16, and *β* = 5.

**Figure 5:**
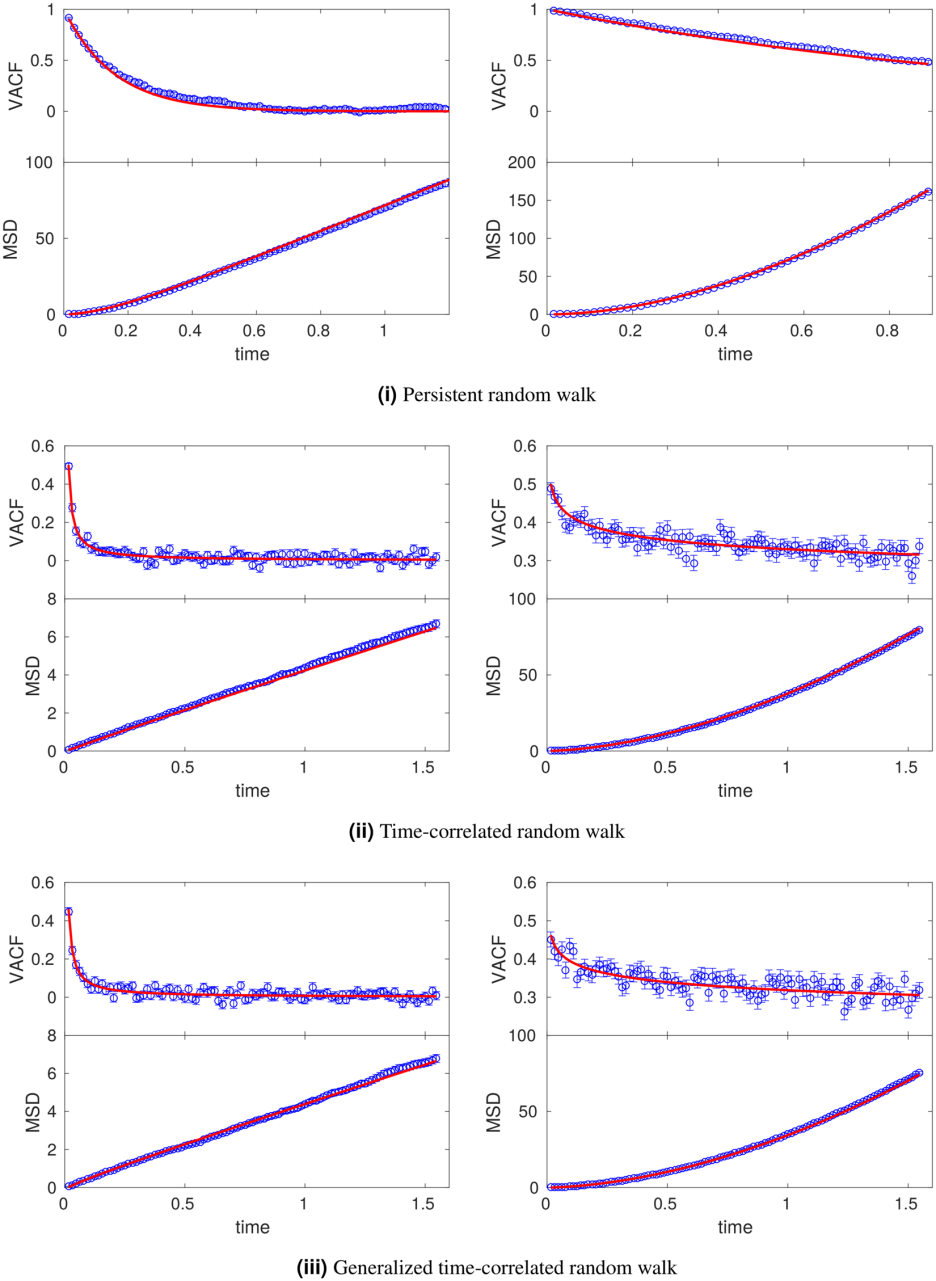
Comparison between persistent, time-correlated and generalized time-correlated random walk models. Shown are simulation results (circles, mean ± standard error of the mean) and theoretical prediction (solid line) for VACF (top row) and MSD (bottom row). Parameters are *v* = 16 and *D*_*rw*_ = 1 in all cases. i) Sensitivity values: *β* = 3 (left), *β* = 5 (right). ii) and iii) *C*_0_ = 0.5, Δ = 0.016, and exponents: *ϕ* = 1 (left), *ϕ* = 0.1 (right).

Comparing Eqs (5) to (12) in Fig. 4 we see that in this second model the velocities are no longer delta correlated but now decay exponentially in time. On the other hand, for long times both Eqs (7) and (15) yield normal diffusion. However, for short times cells performing persistent random walks move superdiffusively, contrary to cells performing classical random walks; see again Fig. 4. In Fig. 5i, Eqs (12) and (15) are compared with results from LGCA simulations, where we see that the theory adequately predicts the observed simulation results. Details on the computational implementation are found in Sec. H of the Supplementary Information.

## Time-correlated random walk

Due to the exponential decay of correlations the previous model did not show superdiffusion at long time scales. It turns out that finding a homogeneous, isotropic Markovian model that shows superdiffusion and power-law decaying correlations is not possible (for a proof see Sec. C of the Supplementary Information).

**Theorem 1** The velocity autocorrelation function of a particle whose orientations are given by a homogeneous, symmetric Markov chain is either delta-correlated, i.e. *g*_*k*_ = *δ*_0,*k*_, where *δ* is the Kronecker delta; alternating, i.e. *g*_*k*_ = (−1)^*k*^ *a* ^*k*^, $a\in {{\mathbb{R}}}^{+}$; or exponentially decaying, i.e. *g*_*k*_ = *e* ^*αk*^, *α* ≤ 0.

To reproduce superdiffusion and power law decaying autocorrelations, we will construct a non-homogeneous model by assuming that the time dependency of the VACF is a known power law.

### Rule derivation

We now assume that the VACF *g*(*t*) is known. In particular, if the movement is power law-correlated the VACF has the form^53^

16 $$g(t)={C}_{0}{(\frac{{\rm{\Delta }}}{t})}^{\varphi },\,t\ge {\rm{\Delta }},$$

where Δ > 0 and 0 < *ϕ* < ∞ and assume that Δ ≪ 1, to disregard the movement at short times, where Eq. (16) diverges. The rate of decay of the VACF is proportional to the exponent *ϕ*. The crossover time Δ specifies the time at which *g*(*t*) = *C*_0_. The walk is positively correlated if *C*_0_ > 0, and anti-correlated if *C*_0_ < 0. Because the process is non-homogeneous, ${P}_{{i}_{k},k}$ in Eq. (3) explicitly depends on the velocity channels ${\overrightarrow{c}}_{i}$ and the current time step *k*. Combining Eqs (3) and (16) we obtain the following relation^47^:

17 $$\sum _{i=1}^{b}{P}_{{i}_{k},k}[{\overrightarrow{c}}_{{i}_{0}}\cdot {\overrightarrow{c}}_{{i}_{k}}]=g(k\mathrm{).}$$

It is possible to derive the reorientation probabilities ${P}_{{i}_{k},k}$ by expanding Eq. (17) for every time step. Additionally, in order to reduce the number of equations, we make the following assumptions:

- The reorientation probabilities are independent, that is, the probability of following a certain trajectory is ${P}_{{i}_{1},{i}_{2},\cdots ,{i}_{k}}={\prod }_{j=1}^{k}{P}_{{i}_{j},j}$.
- There is symmetry around the initial orientation, i.e. if ${\overrightarrow{c}}_{{i}_{k},k}\cdot {\overrightarrow{c}}_{{i}_{0}}={\overrightarrow{c}}_{{j}_{k},k}\cdot {\overrightarrow{c}}_{{i}_{0}}$ then ${P}_{{i}_{k},k}={P}_{{j}_{k},k}$, *i*_*k*_ ≠ *j*_*k*_.

Using these assumptions we can derive the general expression for the reorientation probabilities determining a certain VACF (see Sec. D in the Supplementary Information)

18 $${P}_{{i}_{k},k}=\frac{1+d[{\overrightarrow{c}}_{{i}_{0}}\cdot {\overrightarrow{c}}_{{i}_{k}}]g(k)}{b},$$

where *d* is the dimension of space and *b* is the number of lattice directions given by the lattice geometry. If the VACF follows a power law, then Eq. (18) is always valid if the crossover time Δ is smaller than the time step length, as the divergence of Eq. (16) is avoided. If Δ ≫ 0, we assume that the movement at short times is completely correlated, i.e. the VACF is given by

19 $$g(t)=\{\begin{array}{ll}1 & t\le {t}^{* }\\ {C}_{0}{(\frac{{\rm{\Delta }}}{t})}^{\varphi } & t > {t}^{* }\end{array},$$

where *t* ^*^ is such that ${C}_{0}{(\frac{{\rm{\Delta }}}{{t}^{* }})}^{\varphi }=1$. We can then define a piecewise reorientation probability

20 $${P}_{{i}_{k},k}=\{\begin{array}{ll}\delta ({\overrightarrow{c}}_{{i}_{k}}-{\overrightarrow{c}}_{{i}_{0}}) & k\le \omega \\ \frac{1+d[{\overrightarrow{c}}_{{i}_{0}}\cdot {\overrightarrow{c}}_{{i}_{k}}]g(k)}{b} & k > \omega \end{array},$$

where *ωτ* = *t* ^*^ is the duration of ballistic motion.

### Model analysis and results

For this model the VACF is obviously known as the reorientation probabilities were calculated specifically to reproduce it. We now calculate the MSD for this model. The reorientation probabilities given by Eq. (18) only depend on the initial cell orientation ${\overrightarrow{c}}_{{i}_{0}}$ and on the time step *k*, so they are independent of other orientations at other times. Because of this independence of orientations the MSD is given by (see Sect. A in the Supplementary Information)

21 $$\langle {r}_{k}^{2}\rangle =2d{D}_{rw}[k\tau -2\sum _{i=1}^{k}{g}^{2}(i)\tau ]+2{v}^{2}\sum _{i=1}^{k}\sum _{j=i}^{k}g(i)g(j){\tau }^{2}$$

yielding a Taylor-Green-Kubo formula^47,54^. In the limit of small time step lengths *τ* → 0 the MSD is given by

22 $$\langle {r}_{t}^{2}\rangle =2d{D}_{rw}[t-2{\int }_{{\rm{\Delta }}}^{t}{g}^{2}(\tau ){\rm{d}}\tau ]+2{v}^{2}{\int }_{{\rm{\Delta }}}^{t}{\int }_{\tau }^{t}g(\tau )g(k){\rm{d}}k{\rm{d}}\tau .$$

Equation (22) shows that there is a correction to the random walk diffusion coefficient as well as a new term depending on the particle speed. If *g*(*t*) is given by Eqs (16) and (22) can be integrated yielding

23a $$\begin{array}{rcl}\langle {r}_{t}^{2}\rangle  & = & 2d{D}_{rw}\{t+\frac{2{C}_{0}^{2}{\rm{\Delta }}}{1-2\varphi }[1-{(\frac{{\rm{\Delta }}}{t})}^{2\varphi -1}]\}\\  &  & +\,2{(\frac{v{C}_{0}{\rm{\Delta }}}{\varphi -1})}^{2}[\frac{1}{2}{(\frac{{\rm{\Delta }}}{t})}^{2\varphi -2}-{(\frac{{\rm{\Delta }}}{t})}^{\varphi -1}+\frac{1}{2}],\quad \varphi \ne \mathrm{1,}\varphi \ne \frac{1}{2}\end{array}$$

23b $$\begin{array}{rcl}\langle {r}_{t}^{2}\rangle  & = & 2d{D}_{rw}[t+2{C}_{0}^{2}{\rm{\Delta }}\,\mathrm{ln}(\frac{{\rm{\Delta }}}{t})]\\  &  & +\,2{v}^{2}{C}_{0}^{2}[2t{\rm{\Delta }}+2{{\rm{\Delta }}}^{2}-4{\rm{\Delta }}{({\rm{\Delta }}t)}^{\frac{1}{2}}],\quad \varphi =\frac{1}{2}\end{array}$$

23c $$\begin{array}{rcl}\langle {r}_{t}^{2}\rangle  & = & 2d{D}_{rw}[t+2{C}_{0}^{2}(\frac{{{\rm{\Delta }}}^{2}}{t}-{\rm{\Delta }})]\\  &  & +\,{[v{C}_{0}{\rm{\Delta }}\mathrm{ln}(\frac{{\rm{\Delta }}}{t})]}^{2},\quad \varphi =1.\end{array}$$

These expressions for the MSD are valid when Δ → 0. For long crossover times when reorientation probabilities are given by Eq. (20), the MSD is (see Sec. E in the Supplementary Information):

24 $$\langle {r}_{t}^{2}\rangle =\{\begin{array}{ll}{(vt)}^{2} & t\le {t}^{* },\\ 2d{D}_{rw}[(t-{t}^{* })-2{\int }_{{t}^{* }}^{t}{g}^{2}(\tau ){\rm{d}}\tau ] & \\ +{v}^{2}[2{\int }_{{t}^{* }}^{t}{\int }_{\tau }^{t}g(\tau )g(k){\rm{d}}k{\rm{d}}\tau +{t}^{* 2}+2{t}^{* }{\int }_{{t}^{* }}^{t}g(\tau ){\rm{d}}\tau ] & t > {t}^{* }\mathrm{.}\end{array}$$

Figure 5ii shows a comparison of Eqs (16) and (22) with LGCA simulations. Details on the computational implementation are found in Sec. H of the Supplementary Information. From Eq. (23) we see that in general $\langle {r}_{t}^{2}\rangle \sim t\pm {t}^{1-2\varphi }+{t}^{2(1-\varphi )}-{t}^{1-\varphi }$, which defines three regimes:

1. $\varphi  < \frac{1}{2}$: superdiffusive regime, arising from the term *t* ^2(1−*ϕ*)^
2. $\frac{1}{2} < \varphi  < 1$: subdiffusive regime, as the term *t* ^2(1−*ϕ*)^ dominates at short times only
3. $\varphi  > 1$: normal diffusive regime, as the linear term is the dominating term

**Figure 6:**
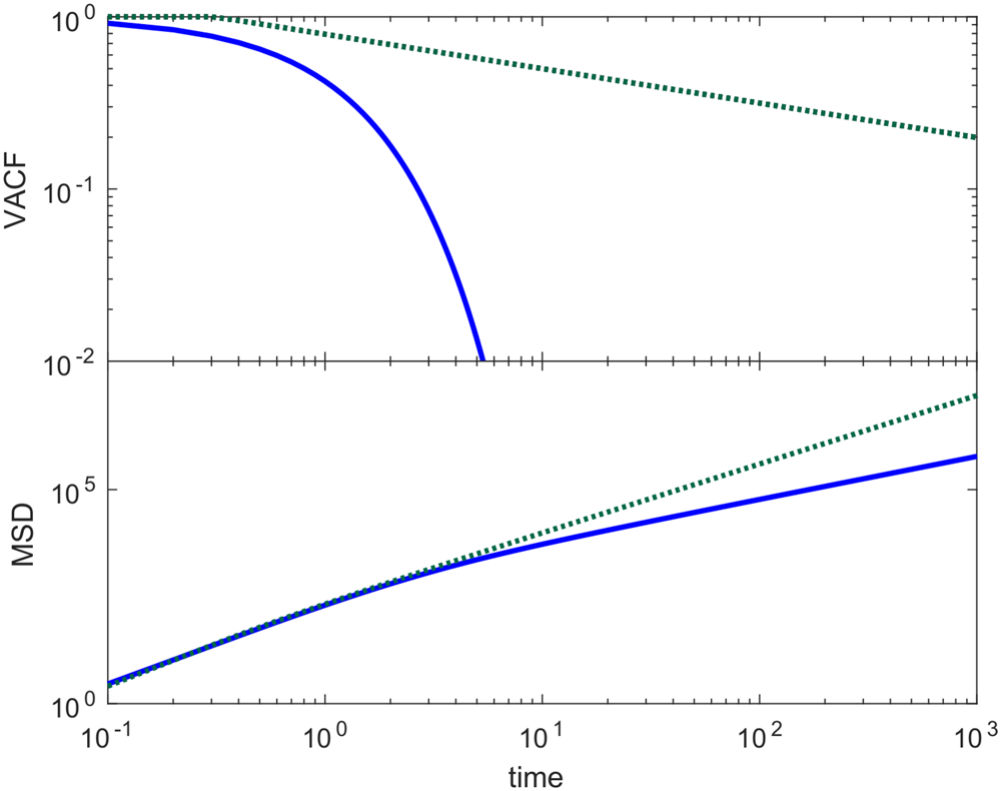
Comparison between persistent and time-correlated random walk models. Shown are theoretical results for VACF (top) and MSD (bottom) in the persistent (solid blue line) and time-correlated (dotted green line) models. The parameter values are *D*_*rw*_ = 1, *v* = 16, *β* = 5, *C*_0_ = 0.5, Δ = 10, and *ϕ* = 0.2.

Figure 6 shows that, while the VACF decays rapidly in the persistent model, in the time-correlated model the VACF decays much more slowly. Additionally, the movement is superdiffusive in both models are short times, however this behavior is long-lasting in the time-correlated model.

## Generalized time-correlated random walk

The reorientation probabilities derived for the time-correlated random walk are only valid for certain time ranges, due to the divergence of the VACF when *t* → 0. We will now derive a generalized model which is valid on both short and long time scales. For this purpose we use what is called the maximum caliber formalism^55^, which we introduce briefly.

### Rule derivation

The maximum caliber formalism has been proven successful to derive models for dynamic systems from data. The procedure consists in maximizing the entropy over a path of system evolutions, with the constraint of reproducing certain observables. The procedure of entropy maximization does not only ensure that the resulting model contains as few assumptions as possible, but is also considered the only method of correctly obtaining unknown probability distributions from known data^55^. The procedure is as follows:

Let the path entropy, or caliber, be defined as ${\mathscr{C}}=-{\sum }_{{\rm{\Gamma }}}{P}_{{\rm{\Gamma }}}\,\mathrm{ln}\,{P}_{{\rm{\Gamma }}},$ where Γ is a possible path followed by the system during its evolution. The probability of following such a path is given by *P*_Γ_. In the case of a single random walker, the path is the entire history of particle velocities Γ = *i*_0_ *i*_1_ *i*_2_ ...*i*_*k*_ up to the last time step *k*. Furthermore, we constrain the unknown probabilities by a normalization constant and the observed VACF (in this case Eq. (17)). Then the problem translates into optimizing the functional^56^ $\tilde{{\mathscr{C}}}[{P}_{{\rm{\Gamma }}}]=-{\sum }_{{\rm{\Gamma }}}{P}_{{\rm{\Gamma }}}{\rm{l}}{\rm{n}}{P}_{{\rm{\Gamma }}}+{\sum }_{j=1}^{k}\beta (j)[{\sum }_{{\rm{\Gamma }}}{P}_{{\rm{\Gamma }}}$ $({\overrightarrow{c}}_{{i}_{0}}\cdot {\overrightarrow{c}}_{{i}_{j}})-g(j)]+\lambda ({\sum }_{{\rm{\Gamma }}}{P}_{{\rm{\Gamma }}}-1)$, where *β*(*j*) and *λ* are Lagrange multipliers to be determined. The Lagrange multiplier *β*(*j*) is given by *β*(*j*) = *dg*(*j*) (see Sec. F in the Supplementary Information), and *λ* determines the normalization constant.

Using the expression for *β*(*j*) we obtain the reorientation probability

25 $${P}_{{i}_{k},k}=\frac{1}{z}\exp [dg(k)({\overrightarrow{c}}_{{i}_{0}}\cdot {\overrightarrow{c}}_{{i}_{k}})],$$

where *z* is the normalization constant for the reorientation probability.

If one required not only that the VACF was observed but also that the autocorrelation function would decay similarly independently of the start and end points, i.e. $\langle {\overrightarrow{c}}_{{i}_{m}}\cdot {\overrightarrow{c}}_{{i}_{n}}\rangle =\langle {\overrightarrow{c}}_{{i}_{j}}\cdot {\overrightarrow{c}}_{{i}_{l}}\rangle$, if *n* − *m* = *l* − *j*, the problem would be similar with the exception that we would now have $\frac{k(k+\mathrm{1)}}{2}$ constraints if the trajectory consists of *k* time steps. Analogously, the probabilites would then be given by

26 $${P}_{{\rm{\Gamma }}}-=\frac{1}{Z}\exp [\sum _{j=1}^{k}\sum _{m=j-1}^{k-1}dg(j-m)({\overrightarrow{c}}_{{i}_{m}}\cdot {\overrightarrow{c}}_{{i}_{j}})]\mathrm{.}$$

### Model analysis and results

The VACF can be easily calculated by using the properties of the partition function *Z*. Given a distribution $P(x)=\frac{1}{Z}\exp [-\beta H(x)]$, the expected value of the function *H*(*x*) is

27 $$\langle H\rangle =-\frac{\partial }{\partial \beta }\,\mathrm{ln}\,Z.$$

Combining Eqs (3), (25) and (27) the VACF is given by $g(k)=\frac{\partial }{\partial \beta (k)}\,\mathrm{ln}\,z$, where *β*(*k*) = *β*_0_(*k*) in the case of of probabilities given by Eq. (26). In the case of a 2D square lattice the partition function can be easily calculated as $z=2\{1+\,\cosh [\beta (k)]\}$. Taking the logarithm and differentiating we obtain $g(k)=\frac{\sinh [\beta (k)]}{1+\,\cosh [\beta (k)]}=\,\tanh [\frac{\beta (k)}{2}]$. If we consider power law correlations and take the limit *τ* → 0 we obtain

28 $$g(t)=\,\tanh [{C}_{0}{(\frac{{\rm{\Delta }}}{t})}^{\varphi }].$$

**Figure 7:**
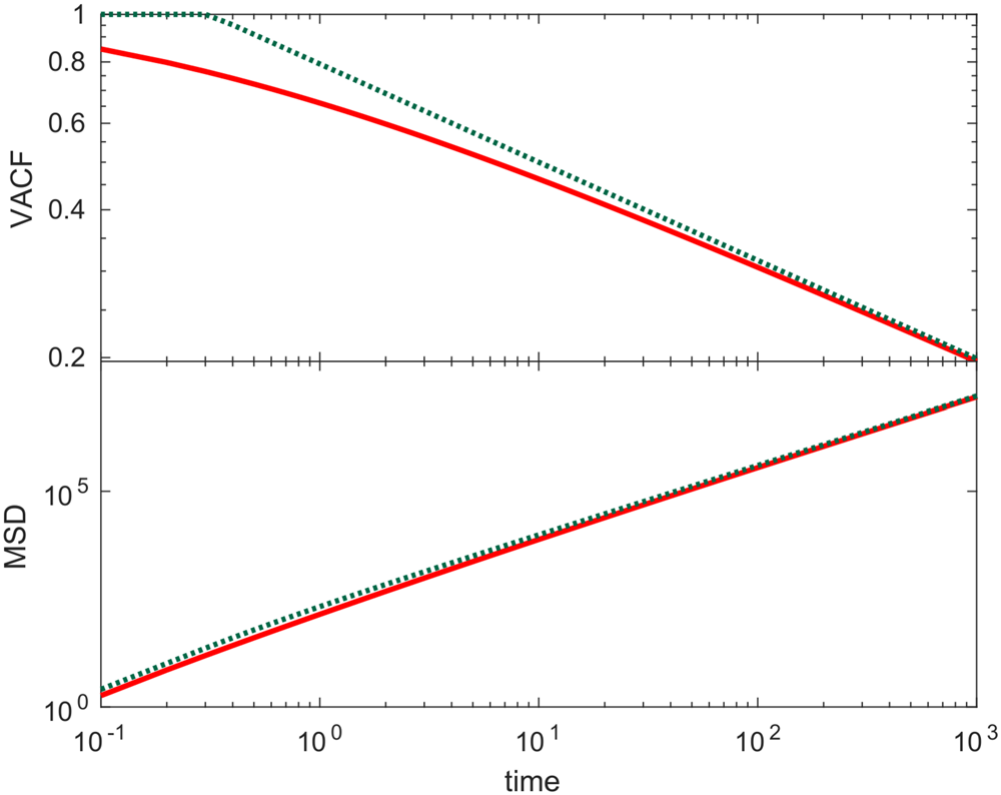
Comparison between generalized and time-correlated random walk models. Shown are theoretical results for VACF (top) and MSD (bottom) in the time-correlated (dotted green line) and generalized time-correlated (solid red line) models. The parameter values are *D*_*rw*_ = 1, *v* = 16, *C*_0_ = 0.5, Δ = 10, *ϕ* = 0.2.

Eqs (16) and (28), corresponding to the time-correlated random walk and generalized time-correlated random walk, respectively, are visually compared in Fig. 7. A Taylor expansion of *g*(*t*) around ${(\frac{{\rm{\Delta }}}{t})}^{\varphi }=0$ (i.e. valid for Δ → 0 or *k* → ∞) shows that up to second order terms $g(t)\approx {C}_{0}{(\frac{{\rm{\Delta }}}{t})}^{\varphi }$, as expected. For ${(\frac{{\rm{\Delta }}}{t})}^{\varphi }\gg 0$ the VACF decays as a power law as well (see Sec. G in the Supplementary Information), $g(t)\approx G(t)=\,\tanh ({C}_{0}){(\frac{{\rm{\Delta }}}{t})}^{2{C}_{0}\varphi {\rm{c}}{\rm{s}}{\rm{c}}{\rm{h}}(2{C}_{0})}$, and the difference between both functions behaves as $g(t)-G(t)\propto {(\frac{\varphi }{{\rm{\Delta }}})}^{2}$. When the probabilites are given by Eq. (25) particle orientations are independent of one another, as it was the case in the time-correlated model, and the MSD is given by Eq. (21). Therefore, in the limit *τ* → 0 the MSD is

29 $$\begin{array}{rcl}\langle {r}_{t}^{2}\rangle  & = & 2d{D}_{rw}\{t-2{\int }_{0}^{t}{\tanh }^{2}[\frac{\beta (\tau )}{2}]{\rm{d}}\tau \}\\  &  & +2{v}^{2}{\int }_{0}^{t}{\int }_{\tau }^{t}\,\tanh [\frac{\beta (\tau )}{2}]\tanh [\frac{\beta (k)}{2}]{\rm{d}}k{\rm{d}}\tau \mathrm{.}\end{array}$$

If we consider power law correlations and expand in Taylor series around Δ^*ϕ*^ = 0 up to second order, we recover Eq. (22) when *g*(*t*) is given by Eq. (16). Therefore the MSD in this model follows similar regimes as those of the time-correlated model Eq. (16). Equations (24) and (29) are visually compared in Fig. 7, and see Fig. 5iii for a comparison of Eqs (28) and (29) with LGCA simulations. Details on the computational implementation are found in Sec. H of the Supplementary Information.

When the probabilities are given by Eq. (26) instead the MSD is given by Eq. (13), as all orientation pairs *i* and *j* are correlated, where the correlation is given by *g*(*i* − *j*) thus not depending specifically on the values of *i* and *j* but on their difference only. Therefore, for a 2D square lattice and a power-law decaying VACF in the limit *τ* → 0 the MSD is given by

30 $$\langle {r}_{t}^{2}\rangle =2d{D}_{rw}t+2{v}^{2}{\int }_{0}^{t}(t-\tau )\tanh [{C}_{0}{(\frac{{\rm{\Delta }}}{\tau })}^{\varphi }]{\rm{d}}\tau \mathrm{.}$$

Again we see that this expression agrees with the formal solution of the MSD for an overdamped Langevin equation with colored noise^53^. In this case, however, the noise correlation is not decaying exponentially. If we expand the hyperbolic tangent on the right hand side around ${(\frac{{\rm{\Delta }}}{t})}^{\varphi }=0$ (i.e. Δ → 0 or *t* → ∞) up to the second term and integrate we obtain the MSD as

31a $$\begin{array}{ll}\langle {r}_{t}^{2}\rangle  & =2d{D}_{rw}t+\frac{2{C}_{0}}{1-\varphi }{(v{\rm{\Delta }})}^{2}\{\frac{1}{2-\varphi }[{(\frac{{\rm{\Delta }}}{t})}^{\varphi -2}-1]+1-\frac{t}{{\rm{\Delta }}}\},\quad \varphi \ne \mathrm{1,}\,\varphi \ne 2\end{array}$$

31b $$\begin{array}{ll}\langle {r}_{t}^{2}\rangle  & =\,2d{D}_{rw}t+2{C}_{0}{(v{\rm{\Delta }})}^{2}\{\frac{t}{{\rm{\Delta }}}[\mathrm{ln}(\frac{t}{{\rm{\Delta }}})-1]+1\},\quad \varphi =1\end{array}$$

31c $$\begin{array}{ll}\langle {r}_{t}^{2}\rangle  & =\,2d{D}_{rw}t+2{C}_{0}{(v{\rm{\Delta }})}^{2}[\frac{t}{{\rm{\Delta }}}-\,\mathrm{ln}(\frac{t}{{\rm{\Delta }}})-1],\quad \quad \quad \varphi =2.\end{array}$$

We conclude from Eq. (31) that

$$\langle {r}_{t}^{2}\rangle \sim t\pm {t}^{2-\varphi }.$$

When *ϕ* < 1 we have 2 − *ϕ* > 1 and the process is superdiffusive. When 1 < *ϕ* < 2 we have 0 < 2 − *ϕ* < 1, at short times the term *t* ^2−*ϕ*^ dominates, and the process is subdiffusive while at long times the linear term dominates yielding normal diffusion. Finally, when *ϕ* > 2 we have 2 − *ϕ* < 0 and the process is completely normal diffusive.

## Summary and Discussion

The goal of our study was to design a simple model for a single particle moving with memory in abscence of any environmental cue. We chose a cellular automaton, specifically, an LGCA because it is a flexible and computationally efficient framework and has the potential to analyze collective behavior in populations of moving particles or cells. After having introduced an LGCA model for unbiased random walk, we have derived three different novel time-correlated LGCA models for single particle migration.

The subsequent persistent random walk LGCA model was derived from a biophysicallymotivated Langevin equation for particle reorientation in an overdamping environment. We showed that in this model the VACF decays exponentially. Furthermore, we proved that a particle in this model moves superdiffusively only at short times while it diffuses normally in the long time limit. This behavior as well as the expression we found for the MSD agree with that found by Othmer^57^ using the telegrapher’s equation and also to the one by Chechkin *et al*.^53^ for exponential noise.

The time-correlated random walk model was derived by assuming that the specific form of the VACF is known. We also assumed that reorientation probabilities were completely independent, and that particles have no preference in turning left or right. We considered the specific case of a power law-decaying VACF and showed that the MSD exhibits two transitions when $g(t)\propto {t}^{-\frac{1}{2}}$ and *g*(*t*) ∝ *t* ^−1^. For small exponents the particle moves superdiffusively on every time scale. At intermediate exponents there are nonlinear contributions dominating at short times. For large exponents, all nonlinear contributions vanish in the long time limit resulting in normal diffusion.

Finally, we derived a generalized LGCA model by maximizing the diffusing particle’s path entropy while retaining the constraint of reproducing a certain VACF. In this model the reorientation probability Eq. (25) is similar to the reorientation probability of the persistent random walk Eq. (10), with some differences: in Eq. (25) the particle’s orientation is compared to its initial orientation while in Eq. (10) the particle’s orientation is compared to the particle’s orientation at the previous time step. Furthermore, in Eq. (10) we have a constant parameter *β* while in Eq. (27) this parameter decays with time, i.e. *β* ∝ *g*(*t*). We recall that Eq. (10) results from considering a Langevin equation for the particle’s reorientation. The parameter *β* depends on the magnitude of the reorienting force, the relaxation constant *γ* (related to friction) and the angular diffusion constant *D*_*θ*_. A time-dependent parameter *β*(*k*) would be obtained when considering a generalized Langevin equation resulting from either a time-dependent reorientation force or friction if these values changed much more slowly than the time needed for the particle to be displaced. Taking this into account, Eq. (10) describes the movement of a particle when reorientations can be performed almost instantaneously compared to the time required for the particle to move in space. Eq. (25) on the other hand describes movement when the particle keeps moving but needs considerably more time to change its initial orientation. When in addition the VACF is required to be invariant under time translations we showed that the corresponding MSD time regimes match to those found by Chechkin *et al*.^53^ for power law-correlated noise.

We have verified our analytical results of all constructed models by comparing them to LGCA computer simulations. In order to derive the analytical VACF and MSD expressions, we have considered the limit *τ* → 0. In this limit, the macroscopic time *t* remains small even after several time steps. It stands to reason that, for *t* → ∞, *τ* ≪ *t*, the difference between our analytical expressions and simulations becomes negligible.

In their present form our LGCA models assume (i) the particle has constant instantaneous speed *v*; (ii) the particle moves to a neighboring site at every time step; and (iii) the particle moves on a regular lattice, which impacts the specific expression of the VACF. All these models could be extended by considering different instantaneous speeds, as well as waiting times between subsequent displacements, by using multispeed LGCA and adding rest (zero velocity) channels, respectively. Effects of the lattice regularity on the single particle movement can be compensated by choosing the sensitivity *β* appropriately in the persistent random walk model as well as the crossover time Δ in the generalized time-correlated model. In the time-correlated model the VACF does not depend on the lattice geometry.

Our new models could also be extended to account for external forces acting on the particle, independent from its intrinsic anomalous movement. For extending the models, we can consider that particle reorientations are caused by internal correlations of individual cells and by particle interactions. The probability ${P}_{{n}^{1},\ldots ,{n}^{N},k}^{corr}$ of having *N* particles in a node with a certain orientation due to internal particle orientations has already been introduced in Eq. (1), while individual particle reorientation probabilities ${P}_{{n}^{\ell },k}$ would be given according to one of the models introduced in this work. On the other hand, the probability ${P}_{{n}^{1},\ldots ,{n}^{N},k}^{int}$ of having *N* particles in a node with a certain orientation due to particle interactions would be a function of other particles’ positions and orientations (see^50^, for examples of such probabilities). If we assume that both probabilities are independent, then the reorientation probability for all particles would simply be ${P}_{{n}^{1},\ldots ,{n}^{N},k}^{tot}={P}_{{n}^{1},\ldots ,{n}^{N},k}^{corr}\cdot {P}_{{n}^{1},\ldots ,{n}^{N},k}^{int}$. Such an extension could be useful for studying physical systems such as plasma gases^14^. Furthermore, it would be interesting to construct LGCA models generating Lévy walks exhibiting non-Gaussian probability density functions^5,8^. More importantly, and tracing back to the original motivation of our work, due to the computational efficiency of LGCAs, our schemes could be applied to model large groups of interacting cells to study the impact of persistence and time correlations in single-cell dynamics on collective phenomena. Highly promising examples are coordination and swarming in bacteria^34^, pluripotent cells during early development^25^, and the emergence of phase transitions in collective cell migration^28–31^. Moreover, the non-cellular microenvironment is crucial for cell migration phenomena. Recently, the impact of complex environments on cell dissemination has been studied with a cellular automaton model^58^. It would be interesting to extend the models introduced here to analyze the impact of anomalous dynamics and complex microenvironments on cell dissemination and cancer invasion.

The LGCA modeling framework followed in this work is characterized by simplifying the concept of a moving particle to movement in discrete time steps between discrete nodes on a regular lattice, possessing only a finite, discrete set of velocities. On one hand, this “discrete approach” is decidedly more simplified and abstract than “continuous approaches” such as continuous time random walks or fractional diffusion equations. On the other hand, as we have shown in the present work, the LGCA offers an advantage not only in computational efficiency and straightforward multiparticle extension, but also in ease of model analysis. This gets rapidly complex in the aforementioned continuous approaches^1–8^ but remains feasible in the LGCA, even when dealing with systems of interacting particles^35,38,42^.

In the era of “Big Data”, there is an abundance of biological data. Single or collective cell motility can be measured *in vitro* or *in vivo* via various experimental methods such as *in vivo* two-photon imaging^59^ or cell cytometry^60^, respectively. In this regard, there is a need for “data-driven” modeling frameworks. Our work comes timely to fulfill this scope by proposing the “data-driven” modeling of single particle superdiffusive behavior without prior knowledge of the mechanisms at work. Such an approach is vital for the study of phenomena whose driving mechanisms are currently unknown or challenging to model^61–63^.

## Electronic supplementary material

Supplementary information
