# Supplementary material for: Cellular automaton models for time-correlated random walks: derivation and analysis
Source: Sci Rep. 2017 Dec 5;7:16952. doi: 10.1038/s41598-017-17317-x (PMC5717221; doi:10.1038/s41598-017-17317-x)
Supplement: Supplementary file 1 — Supplementary information [file 41598_2017_17317_MOESM1_ESM.pdf]

Cellular automaton models for time-correlated random walks: derivation and analysis

J. M. Nava-Sedeño, H. Hatzikirou, R. Klages, A. Deutsch

Supplementary information

MSD in correlated systems

Here we derive the general expression for the MSD when there are temporal correlations. We use the derivation by Schüring[51]. To start with, we consider the "MSD" as defined by Shalchi[52]

$$\langle r_i r_j \rangle = \int_{t_0}^{t} \int_{t_0}^{t} \langle v_i(\tau) v_j(\xi) \rangle \mathrm{d}\xi \mathrm{d}\tau$$

which in LGCA notation are written as

$$\langle r_i r_j \rangle = \sum_{n=1}^{k} \sum_{m=1}^{k} v^2 \tau^2 \langle [\hat{e}_i \cdot \vec{c}_{i_n}] [\hat{e}_j \cdot \vec{c}_{i_m}] \rangle , \tag{S1}$$

where $\hat{e}_x$ and $\hat{e}_y$ are the two orthonormal unit vectors in Cartesian coordinates. In 2D the diagonal elements are given by:

$$\begin{aligned} \langle r_x^2 \rangle &= \sum_{i=1}^{k} \sum_{j=1}^{k} v^2 \tau^2 \langle \cos(\theta_i) \cos(\theta_j) \rangle \\ &= \sum_{i=1}^{k} \sum_{j=1}^{k} \frac{v^2 \tau^2}{2} \langle \cos(\theta_i - \theta_j) + \cos(\theta_i + \theta_j) \rangle \end{aligned} \tag{S2}$$

and

$$\begin{aligned} \langle r_y^2 \rangle &= \sum_{i=1}^{k} \sum_{j=1}^{k} v^2 \tau^2 \langle \sin(\theta_i) \sin(\theta_j) \rangle \\ &= \sum_{i=1}^{k} \sum_{j=1}^{k} \frac{v^2 \tau^2}{2} \langle \cos(\theta_i - \theta_j) - \cos(\theta_i + \theta_j) \rangle , \end{aligned} \tag{S3}$$

where $\theta_k = \arg [\vec{c}_{i_k}]$. Adding them up gives the MSD

$$\langle r^2 \rangle = \langle r_x^2 \rangle + \langle r_y^2 \rangle = \sum_{i=1}^{k} \sum_{j=1}^{k} v^2 \tau^2 \langle \cos(\theta_i - \theta_j) \rangle , \tag{S4}$$

which is just a Taylor-Green-Kubo formula[47,54]. When $i = j$ we have $\cos(\theta_i - \theta_j) = 1$, so by taking these terms out of the sum we get

$$\langle r^2 \rangle = k v^2 \tau^2 + \sum_{i=1}^{k} \sum_{j=1}^{k} v^2 \tau^2 \langle \cos(\theta_i - \theta_j) \rangle (1 - \delta_{ij}). \tag{S5}$$

On the one hand $v^2 \tau^2 = \varepsilon^2$ and on the other $\varepsilon^2 = 2dD\tau$, so using these relations on the first term on the right hand side yields

$$\langle r^2 \rangle = 2dDk\tau + \sum_{i=1}^{k} \sum_{j=1}^{k} v^2 \tau^2 \langle \cos(\theta_i - \theta_j) \rangle (1 - \delta_{ij}). \tag{S6}$$

Because the cosine is an even function $\cos(\theta_i - \theta_j) = \cos(\theta_j - \theta_i)$, which means that we are adding two identical terms for every $i \neq j$ (this condition is already satisfied due to the Kronecker delta). This situation allows us to rewrite the limits of the interior sum if we take care of counting each term twice. Furthermore, using trigonometric identities it is possible to expand the cosine on the right hand side,

$$\begin{aligned} \langle r^2 \rangle = 2dDk\tau &+ 2 \sum_{i=1}^{k} \sum_{j=i}^{k} v^2 \tau^2 \langle \cos(\theta_i) \cos(\theta_j) \rangle (1 - \delta_{ij}) \\ &+ 2 \sum_{i=1}^{k} \sum_{j=i}^{k} v^2 \tau^2 \langle \sin(\theta_i) \sin(\theta_j) \rangle (1 - \delta_{ij}). \end{aligned} \tag{S7}$$

If the orientations of the particle are completely uncorrelated from one another, then it is possible to write the sums on the right as

$$\left\langle r^2\right\rangle = 2dDk\tau + 2\sum_{i=1}^{k}\sum_{j=i}^{k} v^2\tau^2 \left\langle\cos\left(\theta_i\right)\right\rangle \left\langle\cos\left(\theta_j\right)\right\rangle \left(1-\delta_{ij}\right)$$
$$+2\sum_{i=1}^{k}\sum_{j=i}^{k} v^2\tau^2 \left\langle\sin\left(\theta_i\right)\right\rangle \left\langle\sin\left(\theta_j\right)\right\rangle \left(1-\delta_{ij}\right). \tag{S8}$$

If the reorientation probabilities are even functions of the angle θ_k, then we have that $\langle\sin(\theta_i)\rangle = 0$, and so the second sum on the right hand side disappears leaving

$$\left\langle r^2\right\rangle = 2dDk\tau + 2\sum_{i=1}^{k}\sum_{j=i}^{k} v^2\tau^2 \left\langle\cos\left(\theta_i\right)\right\rangle \left\langle\cos\left(\theta_j\right)\right\rangle \left(1-\delta_{ij}\right). \tag{S9}$$

We can choose our coordinate system such that $\theta_0 = 0$ without loss of generality. With this choice of the coordinate system, we can rewrite the MSD as

$$\left\langle r^2\right\rangle = 2dDk\tau + 2\sum_{i=1}^{k}\sum_{j=i}^{k} v^2\tau^2 \left\langle\cos\left(\theta_i-\theta_0\right)\right\rangle \left\langle\cos\left(\theta_j-\theta_0\right)\right\rangle \left(1-\delta_{ij}\right). \tag{S10}$$

Using the definition of the VACF this becomes

$$\left\langle r^2\right\rangle = 2dDk\tau + 2\sum_{i=1}^{k}\sum_{j=i}^{k} v^2\tau^2 g(i)g(j)(1-\delta_{ij}). \tag{S11}$$

Expanding the difference on the right hand side we get

$$\left\langle r^2\right\rangle = 2dDk\tau + 2\sum_{i=1}^{k}\sum_{j=i}^{k} v^2\tau^2 g(i)g(j) - 2\sum_{i=1}^{k}\sum_{j=i}^{k} v^2\tau^2 g(i)g(j)\delta_{ij}. \tag{S12}$$

The last sum on the right hand side can be simplified, so we get

$$\left\langle r^2\right\rangle = 2dDk\tau + 2\sum_{i=1}^{k}\sum_{j=i}^{k} v^2\tau^2 g(i)g(j) - 2\sum_{i=1}^{k} v^2\tau^2 g^2(i). \tag{S13}$$

Finally, using the relation between the intantaneous particle velocity v and the diffusion constant in the random walk limit D_{rw}, and reordering terms, we obtain

$$\left\langle r^2\right\rangle = 2dD_{rw}\left[k\tau - 2\sum_{i=1}^{k} g^2(i)\tau\right] + 2v^2\sum_{i=1}^{k}\sum_{j=i}^{k} g(i)g(j)\tau^2. \tag{S14}$$

VACF and MSD derivation in the persistent random walk

First we analytically derive the expected form of the VACF for a single particle in an LGCA where the lattice is a 2D square lattice.

VACF

As mentioned before, the orientation probability is given by Eq. (10). The VACF is formally defined as $g(t) = \langle\vec{v}_0 \cdot \vec{v}_t\rangle$, where v_0 and v_t are the velocities of the particle at time 0 and t, respectively. Using this definition, we can calculate the VACF of a stochastically moving particle as

$$g(t) = \int P(\vec{v},t)\,(\vec{v}_0\cdot\vec{v})\,\mathrm{d}\vec{v},$$

where $P(\vec{v},t)$ is the probability of the particle having a velocity $\vec{v}$ at time t.

In an LGCA particle velocities are given by the velocity channels they are located in, which belong to a finite set of unit vectors depending on the lattice dimension and geometry. Furthermore, time is also discrete with time steps of length τ such

that at time step k time has elapsed by $k\tau$. We can then rewrite the definition of the velocity autocorrelation in the case of an LGCA in the following way[47]:

$$g(t) = \langle \vec{v}_0 \cdot \vec{v}_t \rangle = \sum_{i=1}^{b} P_{i_k,k} \left[\vec{c}_{i_0} \cdot \vec{c}_{i_k} \right], \tag{S15}$$

where $\vec{c}_{i_0}$ is the orientation of the particle at time step $k = 0$ and $\vec{c}_{i_k}$ is the orientation of the particle at time step k. To calculate the VACF, we start by defining a function as:

$$H = -\vec{c}_{i_{k-1}} \cdot \vec{c}_{i_k}, \tag{S16}$$

where $\vec{c}_{i_k}$ is the particle orientation at time step k. Having defined this function, we can rewrite Eq. (10) as follows:

$$P_{i_k,k} = \frac{e^{-\beta H\left(\vec{c}_{i_k},k\right)}}{Z}, \tag{S17}$$

where the partition function is defined as

$$Z = \sum_{i_k} e^{-\beta H\left(\vec{c}_{i_k},k\right)}. \tag{S18}$$

The expected value of the function is given by

$$\langle H \rangle = \left\langle -\vec{c}_{i_{k-1}} \cdot \vec{c}_{i_k} \right\rangle, \tag{S19}$$

that is, the energy of the system is the single-step correlation. Due to the distribution of the reorientation probabilites the total energy can be calculated by the well-known relation

$$\langle H \rangle = -\frac{\partial}{\partial \beta} \ln Z. \tag{S20}$$

Using the last two equations, we get an expression for the single step correlation:

$$\left\langle \vec{c}_{i_{k-1}} \cdot \vec{c}_{i_k} \right\rangle = \frac{\partial}{\partial \beta} \ln Z. \tag{S21}$$

In this single-particle model, the partition function can be easily calculated. For a 2D square lattice the partition function reads

$$Z = 2\left[1 + \cosh(\beta)\right]. \tag{S22}$$

Substituting Supplementary Eq. (S22) into Supplementary Eq. (S21), we have

$$\begin{aligned} \left\langle \vec{c}_{i_{k-1}} \cdot \vec{c}_{i_k} \right\rangle &= \frac{\partial}{\partial \beta} \left\{ \ln(2) + \ln\left[1 + \cosh(\beta)\right] \right\} \\ &= \frac{\sinh(\beta)}{1 + \cosh(\beta)} = \tanh\left(\frac{\beta}{2}\right). \end{aligned} \tag{S23}$$

The particle orientations $\vec{c}_i$ are normalized vectors. This allows us to rewrite the single step correlation as

$$\left\langle \vec{c}_{i_{k-1}} \cdot \vec{c}_{i_k} \right\rangle = \langle \cos\left(\theta_k - \theta_{k-1}\right) \rangle, \tag{S24}$$

and the VACF as

$$g(k) = \langle \cos\left(\theta_k - \theta_0\right) \rangle, \tag{S25}$$

where $\theta_k = \arg\left[\vec{c}_k\right]$. Supplemenary Eq. (S25) can be rewritten by adding zeroes in the following way:

$$g(k) = \langle \cos\left(\theta_k - \theta_0\right) \rangle = \left\langle \cos \left[\theta_k - \theta_0 + \sum_{i=1}^{k-1} \left(\theta_i - \theta_i\right) \right] \right\rangle,$$

which after rearranging terms, has the form

$$g(k) = \left\langle \cos\left(\sum_{i=1}^{k} \theta_i - \theta_{i-1}\right)\right\rangle . \tag{S26}$$

Using trigonometric identities and the linearity of the expected value operator we can expand this expression to

$$\begin{aligned} g(k) = & \left\langle \prod_{i=1}^{k} \cos\left(\theta_i - \theta_{i-1}\right)\right\rangle \\ & + f\left\{\left\langle \cos\left(\theta_n - \theta_{n-1}\right)\sin\left(\theta_m - \theta_{m-1}\right)\right\rangle\right\}, \end{aligned} \tag{S27}$$

where f is a sum of expected values of products of sines and cosines. Because the model is Markovian, the i-th orientation is only correlated with the $(i-1)$-th orientation. This allows us to write

$$\begin{aligned} g(k) = & \prod_{i=1}^{k} \left\langle \cos\left(\theta_i - \theta_{i-1}\right)\right\rangle \\ & + f\left\{\left\langle \cos\left(\theta_n - \theta_{n-1}\right)\right\rangle \left\langle \sin\left(\theta_m - \theta_{m-1}\right)\right\rangle\right\}. \end{aligned} \tag{S28}$$

Now, because the reorientation probabilities Eq. (10) are even functions with respect to $\theta_k = \arg\left[\vec{c}(k)\right]$, the expected values become

$$\left\langle \sin\left(\theta_m - \theta_{m-1}\right)\right\rangle = 0,$$

which in turn implies

$$f\left\{\left\langle \cos\left(\theta_n - \theta_{n-1}\right)\right\rangle \left\langle \sin\left(\theta_m - \theta_{m-1}\right)\right\rangle\right\} = 0.$$

Using these relations we find that the VACF is given by

$$g(k) = \prod_{i=1}^{k} \left\langle \cos\left(\theta_i - \theta_{i-1}\right)\right\rangle . \tag{S29}$$

Using Supplementary Eqs. (S23) and (S24) in Supplementary Eq. (S29) yields

$$g(k) = \left[\tanh\left(\frac{\beta}{2}\right)\right]^{k}, \tag{S30}$$

which can be written as

$$g(k) = e^{\alpha k}, \tag{S31}$$

if we define the exponent α as

$$\alpha = \ln\left[\tanh\left(\frac{\beta}{2}\right)\right]. \tag{S32}$$

The exponent α depends on the lattice dimension and geometry, as follows:

- In 1D the exponent is given by:

$$\alpha = \ln\left[\tanh\left(\beta\right)\right]. \tag{S33}$$

- In 2D with a triangular lattice the exponent is:

$$\alpha = \ln\left(\frac{e^{\frac{\beta}{2}} - e^{-\beta}}{e^{-\beta} + 2e^{\frac{\beta}{2}}}\right). \tag{S34}$$

- In 2D with a square lattice the exponent is given by:

$$\alpha = \ln\left[\tanh\left(\frac{\beta}{2}\right)\right]. \tag{S35}$$

- In 2D with an hexagonal lattice it takes the form:

$$\alpha = \ln\left[\frac{2\sinh\left(\frac{3\beta}{4}\right)\cosh\left(\frac{\beta}{4}\right)}{\cosh(\beta)+2\cosh\left(\frac{\beta}{2}\right)}\right]. \tag{S36}$$

- With a cubic 3D lattice the exponent reads:

$$\alpha = \ln\left[\frac{\sinh(\beta)}{\cosh(\beta)+2}\right]. \tag{S37}$$

Mean square displacement

To calculate the MSD of particles performing persistent random walks, we start with Supplementary Eqs. (S6) and (S24) and rewrite the sum limits taking into account that the cosine is an even function to obtain

$$\langle r^2\rangle = 2dDk\tau + 2v^2\sum_{n=1}^{k}\sum_{m=n}^{k}\langle\vec{c}_{i_n}\cdot\vec{c}_{i_m}\rangle\,\tau^2(1-\delta_{nm}). \tag{S38}$$

The expected value on the right hand side is the $(m-n)$-step correlation. From the previous VACF calculation we know that in the Markovian model

$$\langle\vec{c}_{i_n}\cdot\vec{c}_{i_m}\rangle := g(m-n) = \left[\tanh\left(\frac{\beta}{2}\right)\right]^{|m-n|}, \tag{S39}$$

which can be substituted in the expression of the MSD to obtain

$$\langle r^2\rangle = 2dDk\tau + 2v^2\sum_{n=1}^{k}\sum_{m=n}^{k} g(m-n)\tau^2(1-\delta_{nm}). \tag{S40}$$

Because the sums on the right hand side only depend on the interval length $\mid n-m\mid$ and not on the specific values of the indices n and m we can replace both sums by a sum over all posible interval lengths. There are $k-j$ ways to divide an interval of k time steps (because the sums start from $n=1$) into intervals of size j. Taking all into account, the MSD becomes

$$\langle r^2\rangle = 2dDk\tau + 2v^2\sum_{j=1}^{k}(k-j)g(j)\tau^2, \tag{S41}$$

which can also be written as

$$\langle r^2\rangle = 2dDk\tau + 2v^2\sum_{j=1}^{k}(k-j)\,e^{j\alpha}\tau^2,$$

where α is given by Supplementary Eq. (S32). We now distribute the two multiplying time steps τ on the second term on the right hand side, and multiply by one the exponent of the exponential function thus leaving it unchanged:

$$\langle r^2\rangle = 2dDk\tau + 2v^2\sum_{j=1}^{k}(k\tau-j\tau)\,e^{\frac{\alpha}{\tau}j\tau}\tau.$$

We now use the definitions of the diffusion coefficient and the particle speed to obtain the following expression for the time step length:

$$\tau = \frac{2dD}{v^2},$$

and use it to substitute for τ on the denominator of the exponent

$$\langle r^2\rangle = 2dDk\tau + 2v^2\sum_{j=1}^{k}(k\tau-j\tau)\,e^{\frac{v^2\alpha}{2dD}j\tau}\tau.$$

VACF in homogeneous Markovian models

We will now consider a general Markovian model for a single moving particle. The model is then a Markov chain of particle orientations, i.e. the particle can transition between different orientations at each time step.

Definition 1. *The state space of the Markov chain is* $\mathscr{E} = \{\vec{c}_0, \vec{c}_{\pm 1}, \cdots, \vec{c}_{\pm n}, \cdots, \vec{c}_N\}$*, where the* $2N$ *different states are given by*

$$\vec{c}_n = \left(\cos\left(\frac{\pi}{n}\right), \sin\left(\frac{\pi}{n}\right)\right),\ n = 1, \cdots, N-1,$$
$$\vec{c}_0 = (1,0),$$
$$\vec{c}_N = (-1,0).$$

Definition 2. *The state space subset* $\mathscr{E}_0$ *is defined as*

$$\mathscr{E}_0 := \left\{\vec{c}_{\pm 1}, \cdots, \vec{c}_{\pm(N-1)}\right\}.$$

If the space is isotropic, then it is reasonable to require that the probability of the particle turning left or right be identical. Furthermore, we assume that the probability of turning does not depend on the specific time step, i.e. that the Markov process is homogeneous.

Definition 3. *The Markov chain is the stochastic process* $\{X(k) : k \in \mathbb{N}\}$ *where the reorientation probabilities are given by*

$$P(X(k+1) = \vec{c}_m \mid X(k) = \vec{c}_n) := P(\theta := \arg(\vec{c}_n, \vec{c}_m)),$$

where $P(\theta) = P(-\theta)$ *for* $0 <\mid \theta \mid < \pi$*, and the initial condition* $X(0) = \vec{c}_0$*.*

We will use the following shorthand notation: $P(0) := p_0$, $P(\frac{\pi}{n}) = P(-\frac{\pi}{n}) := p_n$, and $P(\pi) := p_N$.

Definition 4. *The rotation matrix* $\mathbf{A_n}$ *is given by*

$$\mathbf{A_n} = \begin{pmatrix} \cos\left(\frac{\pi}{n}\right) & -\sin\left(\frac{\pi}{n}\right) \\ \sin\left(\frac{\pi}{n}\right) & \cos\left(\frac{\pi}{n}\right) \end{pmatrix},$$

such that

$$\mathbf{A_n}\begin{pmatrix} 1 \\ 0 \end{pmatrix} = \begin{pmatrix} \cos\left(\frac{\pi}{n}\right) \\ \sin\left(\frac{\pi}{n}\right) \end{pmatrix} = \vec{c}_n$$

and

$$\mathbf{A_n}\begin{pmatrix} \cos(\phi) \\ \sin(\phi) \end{pmatrix} = \begin{pmatrix} \cos\left(\frac{\pi}{n}\right)\cos(\phi) - \sin\left(\frac{\pi}{n}\right)\sin(\phi) \\ \sin\left(\frac{\pi}{n}\right)\cos(\phi) + \cos\left(\frac{\pi}{n}\right)\sin(\phi) \end{pmatrix} = \begin{pmatrix} \cos(\frac{\pi}{n} + \phi) \\ \sin(\frac{\pi}{n} + \phi) \end{pmatrix}.$$

Definition 5. *The velocity autocorrelation function (VACF) is given by*

$$g_k = \langle X(0) \cdot X(k) \rangle = \sum_{\vec{v} \in \mathscr{E}} (\vec{c}_0 \cdot \vec{v}) P^k(\vec{v})$$

Theorem 2. *The velocity autocorrelation function of a particle whose orientations are given by a homogeneous, symmetric Markov chain is either delta-correlated, i.e.* $g_k = \delta_{0,k}$*, where* δ *is the Kronecker delta; alternating, i.e.* $g_k = (-1)^k a^k$*,* $a \in \mathbb{R}^+$*; or exponentially decaying, i.e.* $g_k = e^{\alpha k}$*,* $\alpha \leq 0$*.*

Proof. The proof is by induction.

$$g_1 = \sum_{\vec{v} \in \mathscr{E}} (\vec{c}_0 \cdot \vec{v}) P(\vec{v}) = \sum_{\vec{v} = \vec{c}_0, \vec{c}_N} (\vec{c}_0 \cdot \vec{v}) P(\vec{v}) + \sum_{\vec{v} \in \mathscr{E}_0} (\vec{c}_0 \cdot \vec{v}) P(\vec{v}) = P(0) - P(\pi) + \sum_{i=1}^{N-1} \left[(\vec{c}_0 \cdot \vec{c}_i) P\left(\frac{\pi}{i}\right) + (\vec{c}_0 \cdot \vec{c}_{-i}) P\left(-\frac{\pi}{i}\right)\right]$$
$$p_0 - p_N + \sum_{i=1}^{N-1} \left[\cos\left(\frac{\pi}{i}\right) p_i + \cos\left(-\frac{\pi}{i}\right) p_i\right] = p_0 - p_N + 2\sum_{i=1}^{N-1} \cos\left(\frac{\pi}{i}\right) p_i := a.$$

Using the Chapman-Kolmogorov equation, we can calculate the VACF at the time step $k+1$

$$g_{k+1} = \sum_{\vec{v} \in \mathscr{E}} (\vec{c}_0 \cdot \vec{v}) P^{k+1}(\vec{v}) = \sum_{\vec{v} \in \mathscr{E}} (\vec{c}_0 \cdot \vec{v}) \sum_{\vec{u} \in \mathscr{E}} P^k(\vec{u}) P(\vec{v} \mid \vec{u}) = \sum_{\vec{u} \in \mathscr{E}} P^k(\vec{u}) \sum_{\vec{v} \in \mathscr{E}} (\vec{c}_0 \cdot \vec{v}) P(\vec{v} \mid \vec{u}).$$

We now expand the second sum on the right hand side of the equation

$$
\begin{aligned}
\sum_{\vec{v}\in\mathscr{E}}(\vec{c}_0\cdot\vec{v})P(\vec{v}\mid\vec{u}) =& (\vec{c}_0\cdot\vec{u})p_0-(\vec{c}_0\cdot\vec{u})p_N+\sum_{i=1}^{N-1}\left[(\vec{c}_0\cdot\mathbf{A_i}\vec{u})P(\mathbf{A_i}\vec{u}\mid\vec{u})+(\vec{c}_0\cdot\mathbf{A_{-i}}\vec{u})P(\mathbf{A_{-i}}\vec{u}\mid\vec{u})\right]\\
&=(\vec{c}_0\cdot\vec{u})p_0-(\vec{c}_0\cdot\vec{u})p_N+\sum_{i=1}^{N-1}\left[\begin{pmatrix}\cos\left(\frac{\pi}{i}\right)\\-\sin\left(\frac{\pi}{i}\right)\end{pmatrix}^T\vec{u}p_i+\begin{pmatrix}\cos\left(\frac{\pi}{i}\right)\\\sin\left(\frac{\pi}{i}\right)\end{pmatrix}^T\vec{u}p_i\right]\\
&=(\vec{c}_0\cdot\vec{u})p_0-(\vec{c}_0\cdot\vec{u})p_N+\sum_{i=1}^{N-1}\begin{pmatrix}2\cos\left(\frac{\pi}{i}\right)\\0\end{pmatrix}^T\vec{u}p_i\\
&=(\vec{c}_0\cdot\vec{u})p_0-(\vec{c}_0\cdot\vec{u})p_N+2\sum_{i=1}^{N-1}\cos\left(\frac{\pi}{i}\right)(\vec{c}_0\cdot\vec{u})p_i\\
&=(\vec{c}_0\cdot\vec{u})\left[p_0-p_N+2\sum_{i=1}^{N-1}\cos\left(\frac{\pi}{i}\right)p_i\right]=(\vec{c}_0\cdot\vec{u})a.
\end{aligned}
$$

Inserting this expression back into the VACF yields

$$g_{k+1}=\sum_{\vec{u}\in\mathscr{E}}P^k(\vec{u})\sum_{\vec{v}\in\mathscr{E}}(\vec{c}_0\cdot\vec{v})P(\vec{v}\mid\vec{u})=\sum_{\vec{u}\in\mathscr{E}}P^k(\vec{u})(\vec{c}_0\cdot\vec{u})a=g_ka=a^ka=a^{(k+1)}.$$

We can rewrite a as $a=\sum_\theta p(\theta)\cos\theta$, where $\theta=\arg(\vec{c}_0,\vec{c}_n)$, $\forall\vec{c}_n\in\mathscr{E}$. Using the fact that $0\leq p(\theta)\leq 1$ and $\sum_\theta p(\theta)=1$, we have

$$-1\leq\cos(\theta)\leq 1\implies -p(\theta)\leq p(\theta)\cos(\theta)\leq p(\theta)\implies -1\leq\sum_\theta p(\theta)\cos\theta\leq 1\therefore -1\leq a\leq 1.$$

We have three cases:

- $-1\leq a<0$, then $a=-1\mid a\mid$ and $g_k=a^k=(-1)^k\mid a\mid^k$.
- $a=0$, then $g_k=a^k=0$, $k\neq 0$.
- $0<a\leq 1$ then $g_k=a^k=e^{k\ln(a)}=e^{\alpha k}$, where $\alpha=\ln(a)$. $0<a\leq 1\implies -\infty<\alpha\leq 0$.

□

Time correlated random walk: rule derivation for different dimensions and geometries

One dimension

We will now sketch our method for obtaining the reorientation probabilities $P_{i_k,k}$ in 1D. We start by expanding $g(k)$ for the first two time steps after $k\tau=t\geq\Delta$ (see Eq. (16)) for a 1D lattice. We will denote by the subscript f the lattice direction parallel to the original orientation of the particle. Similarly, the subscript r denotes the direction opposite to the original orientation of the particle. Numerical subscripts denote the time step at which the reorientation probability is evaluated.

Time step $k=1$ Only two trajectories are possible after one time step. Their probabilities are given by $P_{f,1}$ and $P_{r,1}$. The normalization condition for these probabilties reads

$$P_{f,1}+P_{r,1}=1. \tag{S42}$$

We now expand the VACF:

$$\sum_{i=1}^{1}P_{i_k,k}\left[\vec{c}_{i_0}\cdot\vec{c}_{i_k}\right]=P_{f,1}-P_{r,1}=g(1). \tag{S43}$$

We can substitute $P_{r,1}$ from Supplementary Eq. (S42) into Supplementary Eq. (S43)

$$P_{f,1}-P_{r,1}=P_{f,1}-(1-P_{f,1})=2P_{f,1}-1=g(1).$$

Rearranging terms we obtain the probability for having the same orientation as originally to

$$P_{f,1}=\frac{1+g(1)}{2}. \tag{S44}$$

Substituting Supplementary Eq. (S44) into Supplementary Eq. (S42) we obtain the probability for the particle to turn around after the first time step,

$$P_{r,1} = \frac{1-g(1)}{2}. \tag{S45}$$

After inspection of Supplementary Eqs. (S44) and (S45), and recalling that in the 1D lattice $c_1 = 1$ and $c_2 = -1$, both probabilities can be written as a single expression,

$$P_{i_1,1} = \frac{1+\left[c_{i_0}\cdot c_{i_1}\right] g(1)}{2}, \tag{S46}$$

where i is a placeholder variable for either f or r.

Time step $k=2$ After two time steps, we have four different possible paths for the particle, with four different probabilities. If we assume the probabilities at each time can be written as $P_{ff} = P_{f,1}P_{f,2}$, we can expand the VACF to obtain:

$$P_{f,1}P_{f,2} - P_{f,1}P_{r,2} + P_{r,1}P_{f,2} - P_{r,1}P_{r,2} = \left(P_{f,2} - P_{r,2}\right)\left(P_{f,1} + P_{r,1}\right) = g(2),$$

which by employing Supplementary Eq. (S42) can simplified to:

$$P_{f,2} - P_{r,2} = g(2). \tag{S47}$$

Given that the probabilities in the previous time step were normalized, it is sufficient to require that the probabilities in the current time step be normalized:

$$P_{f,2} + P_{r,2} = 1. \tag{S48}$$

Inspecting Supplementary Eqs. (S47) and (S48) and comparing them with Supplementary Eqs. (S42) and (S43) we can see that they are identical except for the evaluation of $g(k)$. Therefore, for the second time step it holds that

$$P_{i_2,2} = \frac{1+\left[c_{i_0}\cdot c_{i_2}\right] g(2)}{2}. \tag{S49}$$

Any k It is easy to see that for further times we can always assume that the probabilities are uncorrelated so that only the last orientation in the particle's orientation history is relevant for the calculation. If we do, Supplementary Eqs. (S46) and (S49) can be generalized for any time step k in the following way:

$$P_{i_k,k} = \frac{1+\left[c_{i_0}\cdot c_{i_k}\right] g(k)}{2}. \tag{S50}$$

Two dimensions: Triangular lattice

We will repeat the calculation we did in 1D now in 2D for two different lattice geometries to identify possible dependencies on the lattice dimension and/or geometry.

Time step $k=1$ We have three possible lattice directions with lattice vectors given by either $\vec{c_1} = (1,0)$, $\vec{c_2} = \left(-\frac{1}{2}, \frac{\sqrt{3}}{2}\right)$, $\vec{c_3} = \left(-\frac{1}{2}, -\frac{\sqrt{3}}{2}\right)$, or $\vec{c_1} = \left(\frac{1}{2}, \frac{\sqrt{3}}{2}\right)$, $\vec{c_2} = (-1,0)$, $\vec{c_3} = \left(\frac{1}{2}, -\frac{\sqrt{3}}{2}\right)$ on alternating nodes. In the first time step there are three possible paths given by $P_{r,1}$, $P_{a,1}$, and $P_{u,1}$, where $P_{r,1}$ is the probability to reverse orientation. The normalization condition is, in this case, given by

$$P_{r,1} + P_{u,1} + P_{a,1} = 1, \tag{S51}$$

while the VACF is given by

$$\frac{1}{2}\left(P_{u,1} + P_{a,1}\right) - P_{r,1} = g(1). \tag{S52}$$

We need to make an assumption to continue, as there are more variables than equations. We assume that the probability of turning left or right is identical,

$$P_{u,1} = P_{a,1} \coloneqq P_{f,1}. \tag{S53}$$

Under this assumption we can rewrite Supplementary Eq. (S51) as

$$P_{r,1} + 2P_{f,1} = 1, \tag{S54}$$

and Supplementary Eq. (S52) as

$$P_{f,1} - P_{r,1} = g(1). \tag{S55}$$

Substituting $P_{f,1}$ from Supplementary Eq. (S55) into Supplementary Eq. (S54) we obtain

$$P_{r,1} + 2(P_{r,1} + g(1)) = 3P_{r,1} + 2g(1) = 1,$$

which, after rearranging, gives the expression for the probability of the particle to go back:

$$P_{r,1} = \frac{1 - 2g(1)}{3}. \tag{S56}$$

Using Supplementary Eq. (S56) in Supplementary Eq. (S54) we obtain the probability

$$P_{f,1} = \frac{1 + g(1)}{3}. \tag{S57}$$

Examining Supplementary Eqs. (S53), (S56) and (S57) we can summarize them as

$$P_{i_1,1} = \frac{1 + 2\left[\vec{c}_{i_0} \cdot \vec{c}_{i_1}\right] g(1)}{3}. \tag{S58}$$

Time step $k = 2$ In this case there are 9 different possible orientation histories with 9 different probabilities. If we now denote by f and r the lattice directions parallel and antiparallel to the original particle orientation, respectively, and by u and a the remaining lattice directions and assume that the probabilities are uncorrelated, we require that probabilites at the present time step are normalized:

$$P_{u,2} + P_{a,2} + P_{f,2} = 1, \tag{S59}$$

while the VACF has the form

$$\begin{aligned} &P_{u,1}P_{f,2} - \frac{1}{2}P_{u,1}P_{u,2} - \frac{1}{2}P_{u,1}P_{a,2} + P_{a,1}P_{f,2} \\ &- \frac{1}{2}P_{a,1}P_{u,2} - \frac{1}{2}P_{a,1}P_{a,2} + P_{r,1}P_{f,2} - \frac{1}{2}P_{r,1}P_{u,2} - \frac{1}{2}P_{r,1}P_{a,2} \\ &= \left[P_{f,2} - \frac{1}{2}\left(P_{u,2} + P_{a,2}\right)\right]\left(P_{u,1} + P_{a,1} + P_{r,1}\right) = g(2), \end{aligned}$$

which, by Supplementary Eq. (S51), is simplified to:

$$P_{f,2} - \frac{1}{2}\left(P_{u,2} + P_{a,2}\right) = g(2). \tag{S60}$$

To continue, we impose the isotropy condition Supplementary Eq. (S53) denoting by $P_{r,2}$ the probabilities $P_{u,2}$ and $P_{a,2}$. With these assumptions the normalization condition reads

$$P_{f,2} + 2P_{r,2} = 1, \tag{S61}$$

while the VACF is now

$$P_{f,2} - P_{r,2} = g(2). \tag{S62}$$

Inserting $P_{f,2}$ from Supplementary Eq. (S61) into Supplementary Eq. (S62) we obtain the probability $P_{r,2}$:

$$P_{r,2} = \frac{1 - g(2)}{3} \tag{S63}$$

and, using the normalization condition Supplementary Eq. (S61) we obtain the probability $P_{f,2}$:

$$P_{f,2} = \frac{1 + 2g(2)}{3}. \tag{S64}$$

These probabilities can be written in the general form

$$P_{i_2,2} = \frac{1 + 2\left[\vec{c}_{i_0} \cdot \vec{c}_{i_2}\right] g(2)}{3}. \tag{S65}$$

Any k From Supplementary Eqs. (S58) and (S65) we can see that for any further time step k and making the same assumptions as before the probabilities are given by

$$P_{i_k,k} = \frac{1+2\left[\vec{c}_{i_0}\cdot\vec{c}_{i_k}\right]g(k)}{3}. \tag{S66}$$

Two dimensions: Square lattice

Time step $k=1$ There are four possible lattice directions with lattice vectors $\vec{c}_1=(1,0)$, $\vec{c}_2=(0,1)$, $\vec{c}_3=(-1,0)$, and $\vec{c}_4=(0,-1)$. Therefore there are four possible probabilities, so the normalization condition reads

$$P_{r,1}+P_{f,1}+P_{u,1}+P_{a,1}=1, \tag{S67}$$

where $P_{u,1}$ and $P_{a,1}$ are the probabilities of going in the two directions orthogonal to the original orientation of the particle. We now expand the VACF to obtain

$$P_{f,1}-P_{r,1}=g(1). \tag{S68}$$

Right from the start we have more variables than equations, so we need to make one more assumption in order to continue with the derivation. To simplify we assume the following:

$$P_{u,1}=P_{a,1}\coloneqq\frac{1}{4}. \tag{S69}$$

With this assumption the normalization condition becomes

$$P_{f,1}+P_{r,1}=\frac{1}{2}. \tag{S70}$$

Inserting Supplementary Eq. (S70) into Supplementary Eq. (S68) we obtain

$$P_{f,1}-(\frac{1}{2}-P_{f,1})=2P_{f,1}-\frac{1}{2}=g(1).$$

Rearranging terms we obtain the probability

$$P_{f,1}=\frac{1+2g(1)}{4}. \tag{S71}$$

Inserting Supplementary Eq. (S71) into Supplementary Eq. (S70) we obtain the remaining probability

$$P_{r,1}=\frac{1-2g(1)}{4}. \tag{S72}$$

Supplementary Equations (S69), (S71) and (S72) can then be summarized as

$$P_{i_1,1}=\frac{1+2\left[\vec{c}_{i_0}\cdot\vec{c}_{i_1}\right]g(1)}{4}. \tag{S73}$$

Time step $k=2$ There are now 16 different possible histories for the traveling particle. As before we assume that the probabilities are uncorrelated which, together with Supplementary Eq. (S67), allows us to write the normalization condition as

$$P_{r,2}+P_{f,2}+P_{u,2}+P_{a,2}=1, \tag{S74}$$

and the VACF now is

$$\begin{aligned}&P_{f,1}P_{f,2}+P_{u,1}P_{f,2}+P_{r,1}P_{f,2}+P_{a,1}P_{f,2}-\\&(P_{f,1}P_{r,2}+P_{u,1}P_{r,2}+P_{r,1}P_{r,2}+P_{a_1}P_{r,2})=\\&\left(P_{f,2}-P_{r,2}\right)\left(P_{f,1}+P_{u,1}+P_{r,1}+P_{a,1}\right)=g(2),\end{aligned}$$

which, by using Supplementary Eq. (S67), is simplified to

$$P_{f,2}-P_{r,2}=g(2). \tag{S75}$$

We see that Supplementary Eqs. (S67) and (S74), and (S68) and (S75) are practically identical. Therefore, by making the same assumptions, we arrive at the following expression for the probabilities at $k=2$:

$$P_{i_2,2}=\frac{1+2\left[\vec{c}_{i_0}\cdot\vec{c}_{i_2}\right]g(2)}{4}. \tag{S76}$$

Any k If we continue making the assumptions we have done until now, the probabilities can be generalized in a straightforward way as

$$P_{i_k,k} = \frac{1+2\left[\vec{c}_{i_0}\cdot\vec{c}_{i_k}\right]g(k)}{4}. \tag{S77}$$

Three dimensions: cubic lattice

Time step $k=1$ In this case we have six lattice directions given by $\vec{c}_1=(1,0,0)$, $\vec{c}_2=(0,1,0)$, $\vec{c}_3=(0,0,1)$, $\vec{c}_4=(-1,0,0)$, $\vec{c}_5=(0,-1,0)$, and $\vec{c}_6=(0,0,-1)$. We denote the lattice direction parallel to the initial orientation with the subindex f, the contrary direction by r and the rest by u, a, d, and s. The normalization condition is

$$P_{f,1}+P_{u,1}+P_{r,1}+P_{d,1}+P_{a,1}+P_{s,1}=1. \tag{S78}$$

The VACF is given by

$$P_{f,1}-P_{r,1}=g(1). \tag{S79}$$

Similarly as in the case of the square lattice, we impose the following condition which allows deriving the reorientation probabilities:

$$P_{u,1}=P_{a,1}=P_{s,1}=P_{d,1}=\frac{1}{6}, \tag{S80}$$

which enables us to simplify the normalization condition in the following way:

$$P_{f,1}+P_{r,1}=\frac{1}{3}. \tag{S81}$$

Using Supplementary Eq. (S81) to substitute $P_{r,1}$ into Supplementary Eq. (S79) we obtain

$$P_{f,1}-(\frac{1}{3}-P_{f,1})=2P_{f,1}-\frac{1}{3}=g(1),$$

which, after rearranging terms yields the probability

$$P_{f_1}=\frac{1+3g(1)}{6}. \tag{S82}$$

Now, using Supplementary Eq. (S81) we can obtain the remaining probability

$$P_{r,1}=\frac{1-3g(1)}{6}. \tag{S83}$$

Examining Supplementary Eqs. (S80), (S82) and (S83) we arrive at the general expression

$$P_{i_1,1} = \frac{1+3\left[\vec{c}_{i_0}\cdot\vec{c}_{i_1}\right]g(1)}{6}. \tag{S84}$$

Any k As done before we can continue the process for further times and, making the same assumptions, we arrive at an equation as Supplementary Eq. (S84) for any time k:

$$P_{i_k,k} = \frac{1+3\left[\vec{c}_{i_0}\cdot\vec{c}_{i_k}\right]g(k)}{6}. \tag{S85}$$

Any dimension, any lattice geometry, any time

Now that probabilities were derived for several dimensions, geometries, and times, we can see from Supplementary Eqs. (S50), (S66), (S77) and (S85) that the general form of the probabilities is given by

$$P_{i_k,k} = \frac{1+d\left[\vec{c}_{i_0}\cdot\vec{c}_{i_k}\right]g(k)}{b}, \tag{S86}$$

where d is the spatial dimension and b is the number of nearest neighbors.

MSD of the piecewise process

We will now calculate the MSD using probabilities such that the VACF is a power-law decaying piecewise function defined as

$$g(t) = \begin{cases} 1 & t \leq t^\star \\ C_0\left(\frac{\Delta}{t}\right)^\phi & t > t^\star \end{cases}, \tag{S87}$$

where $t^\star$ is such that $C_0\left(\frac{\Delta}{t^\star}\right)^\phi = 1$. It is straightforward to see that the probabilities which define such a VACF obey

$$P_{i_k,k} = \begin{cases} \delta_{i,i_0} & k \leq \omega \\ \frac{1+d\left[\vec{c}_{i_0}\cdot\vec{c}_{i_k}\right]g(k)}{b} & k > \omega \end{cases} \tag{S88}$$

where i_0 is the index of the velocity channel the particle started in and ω is such that $t^\star = \omega\tau$. It is easy to see that in the first ω time steps the MSD is defined by

$$\left\langle r^2 \right\rangle(k) = k^2\varepsilon^2,$$

or, using the definition of the particle speed and taking the limit $\tau \to 0$:

$$\left\langle r^2 \right\rangle(t) = (vt)^2. \tag{S89}$$

We will now calculate the MSD for time steps greater than ω. The calculation will be made for a 1D lattice, but the results are identical for any dimension and lattice geometry. To ease notation, we will omit any subindices refering to time steps $k \leq \omega$, as we know that, given Supplementary Eq. (S88), only those trajectories where the first ω orientations of the particle are identical to the original orientation of the particle have non-zero probabilities.

$\omega+1$ At the first time step after ω time steps have elapsed, we find that the MSD is given by

$$\left\langle r^2 \right\rangle(\omega+1) = r_f^2 P_{f,\omega+1} + r_r^2 P_{r,\omega+1},$$

where the displacements are $r_f^2 = (\omega+1)^2\varepsilon^2$ and $r_r^2 = (\omega-1)^2\varepsilon^2$. Using Supplementary Eq. (S88) and substituting the square displacements we obtain

$$\begin{aligned}\left\langle r^2 \right\rangle(\omega+1) &= (\omega+1)^2\varepsilon^2\left[\frac{1+g(\omega+1)}{2}\right] + (\omega-1)^2\varepsilon^2\left[\frac{1-g(\omega+1)}{2}\right] \\ &= \frac{\varepsilon^2}{2}\left\{(\omega^2+2\omega+1)[1+g(\omega+1)] + (\omega^2-2\omega+1)[1-g(\omega+1)]\right\} \\ &= \frac{\varepsilon^2}{2}\left\{2\omega^2+2+2\omega[1+g(\omega+1)-1+g(\omega+1)]\right\}\end{aligned}$$

which reduces to

$$\left\langle r^2 \right\rangle(\omega+1) = \varepsilon^2[\omega^2+1+2\omega g(\omega+1)]. \tag{S90}$$

$\omega+2$ Now, the MSD can be expanded in the following way:

$$\begin{aligned}\left\langle r^2 \right\rangle(\omega+2) = \varepsilon^2 \Big\{ &(\omega+2)^2\left[\frac{1+g(\omega+1)}{2}\right]\left[\frac{1+g(\omega+2)}{2}\right] \\ &+ \omega^2\left[\frac{1+g(\omega+1)}{2}\right]\left[\frac{1-g(\omega+2)}{2}\right] \\ &+ \omega^2\left[\frac{1-g(\omega+1)}{2}\right]\left[\frac{1+g(\omega+2)}{2}\right] \\ &+(\omega-2)^2\left[\frac{1-g(\omega+1)}{2}\right]\left[\frac{1-g(\omega+2)}{2}\right]\Big\} \\ &\frac{\varepsilon^2}{4}\left\{(\omega^2+4\omega+4)[1+g(\omega+1)+g(\omega+2)+g(\omega+1)g(\omega+2)]\right. \\ &+ \omega^2[1+g(\omega+1)-g(\omega+2)-g(\omega+1)g(\omega+2)] \\ &+ \omega^2[1-g(\omega+1)+g(\omega+2)-g(\omega+1)g(\omega+2)] \\ &\left.+(\omega^2-4\omega+4)[1-g(\omega+1)-g(\omega+2)+g(\omega+1)g(\omega+2)]\right\},\end{aligned}$$

which reduces to:

$$\left\langle r^2 \right\rangle(\omega+2) = \varepsilon^2\left\{\omega^2+2+2g(\omega+1)g(\omega+2)+2\omega[g(\omega+1)+g(\omega+2)]\right\}. \tag{S91}$$

Any k We can proceed for further k and will arrive at the following expression for any $k > \omega$:

$$\begin{aligned}\left\langle r^2\right\rangle(\omega+k) = \varepsilon^2 \Bigg[&\omega^2 + k + 2\sum_{i=1}^{k}\sum_{j=i}^{k} g(\omega+i)g(\omega+j) \\ &-2\sum_{i=1}^{k} g^2(\omega+1) + 2\omega\sum_{i=1}^{k} g(\omega+i)\Bigg]\end{aligned} \tag{S92}$$

which by using the definition of the diffusion coefficient and particle speed can be converted to

$$\begin{aligned}\left\langle r^2\right\rangle(\omega+k) = 2dD\left[k\tau - 2\sum_{i=1}^{k} g^2(\omega+i)\tau\right] + v^2\Bigg[&2\sum_{i=1}^{k}\sum_{j=i}^{k} g(\omega+i)g(\omega+j)\tau^2 \\ &+(\omega\tau)^2 + 2\omega\tau\sum_{i=1}^{k} g(\omega+i)\tau\Bigg]\end{aligned}$$

which in the limit $\tau \to 0$ is

$$\begin{aligned}\left\langle r^2\right\rangle(t) = &2dD\left[(t-t^\star) - 2\int_{t^\star}^{t} g^2(\tau)\mathrm{d}\tau\right] \\ &+ v^2\left[2\int_{t^\star}^{t}\int_{\tau}^{t} g(\tau)g(k)\mathrm{d}k\mathrm{d}\tau + t^{\star 2} + 2t^\star\int_{t^\star}^{t} g(\tau)\mathrm{d}\tau\right].\end{aligned} \tag{S93}$$

Combining Supplementary Eqs. (S89) and (S93) we obtain the MSD of a particle with a piecewise power-law decaying VACF:

$$\left\langle r^2\right\rangle(t) = \begin{cases}(vt)^2 & t \le t^\star, \\ 2dD\left[(t-t^\star) - 2\int_{t^\star}^{t} g^2(\tau)\mathrm{d}\tau\right] & \\ \quad + v^2\left[2\int_{t^\star}^{t}\int_{\tau}^{t} g(\tau)g(k)\mathrm{d}k\mathrm{d}\tau + t^{\star 2} + 2t^\star\int_{t^\star}^{t} g(\tau)\mathrm{d}\tau\right] & t > t^\star.\end{cases} \tag{S94}$$

Generalized time-correlated random walk: rule derivation

We maximize the caliber

$$\mathscr{C} = -\sum_{\Gamma} P_\Gamma \ln P_\Gamma, \tag{S95}$$

subject to observing a certain VACF, which translates into the Lagrange multiplier problem

$$\tilde{\mathscr{C}}[P_\Gamma] = -\sum_{\Gamma} P_\Gamma \ln P_\Gamma + \sum_{i=1}^{k}\beta(i)\left[\sum_{\Gamma} P_\Gamma\left(\vec{c}_{n_0}\cdot\vec{c}_{n_i}\right) - g(i)\right] + \lambda\left(\sum_{\Gamma} P_\Gamma - 1\right), \tag{S96}$$

This yields the trajectory probabilites

$$P_\Gamma = \frac{1}{Z}\exp\left[\sum_{i=1}^{k}\beta(i)\left(\vec{c}_{n_0}\cdot\vec{c}_{n_i}\right)\right], \tag{S97}$$

where $Z = \exp(1-\lambda)$ is called the dynamical partition function which, by optimizing the functional with respect to λ (i.e., $\frac{\partial\mathscr{C}}{\partial\lambda} = 0$), is given by $Z = \sum_\Gamma \exp\left[\sum_{i=1}^{k}\beta(i)\left(\vec{c}_{n_0}\cdot\vec{c}_{n_i}\right)\right]$. Optimizing with respect to $\beta(i)$ yields our original constraint

$$g(k) = \sum_{\Gamma} P_\Gamma\left(\vec{c}_{n_0}\cdot\vec{c}_{n_i}\right). \tag{S98}$$

Solving for $\beta(i)$ using Supplementary Eqs. (S97) and (S98) can be quite challenging, so we expand P_Γ in a Taylor series around $\beta(i) = 0$, which reduces to $g(k) \approx \sum_\Gamma \frac{1}{Z}\left[1 + \sum_{i=0}^{k}\beta(i)\left(\vec{c}_{n_0}\cdot\vec{c}_{n_i}\right)\right]\left(\vec{c}_{n_0}\cdot\vec{c}_{n_k}\right)$, where the dynamical partition function is

simplified as

$$Z \approx \sum_{\Gamma} \left[1 + \sum_{i=0}^{k} \beta(i) \left(\vec{c}_{n_0} \cdot \vec{c}_{n_i} \right) \right] = b^k + \sum_{\Gamma} \sum_{i=1}^{k} \beta(i) \left(\vec{c}_{n_0} \cdot \vec{c}_{n_i} \right) = b^k + \sum_{i=1}^{k} \beta(i) \sum_{\Gamma} \left(\vec{c}_{n_0} \cdot \vec{c}_{n_i} \right)$$
$$= b^k + \sum_{i=1}^{k} \beta(i) \sum_{\Gamma} \cos\theta_i = b^k,$$

where b is the number of lattice directions and θ_i is the angle between the original particle orientation and the particle orientation at time step i. $\sum_{\Gamma} \cos\theta_i = 0$ because the lattice directions and hence the possible particle orientations are symmetrically and homogeneously distributed. Substituting Z, using the same notation as previously, employing trigonometric identities, and denoting the spatial dimension by d, we proceed with the calculation:

$$g(k) \approx b^{-k} \left[\sum_{\Gamma} \cos\theta_k + \sum_{\Gamma} \sum_{i=1}^{k} \beta(i) \cos\theta_i \cos\theta_k \right] = b^{-k} \sum_{\Gamma} \sum_{i=1}^{k} \beta(i) \cos\theta_i \cos\theta_k = b^{-k} \sum_{i=1}^{k} \sum_{\Gamma} \beta(i) \cos\theta_i \cos\theta_k$$
$$= b^{-k} \left[\beta(1) \sum_{\Gamma} \cos\theta_1 \cos\theta_k + \beta(2) \sum_{\Gamma} \cos\theta_2 \cos\theta_k + \cdots + \beta(k-1) \sum_{\Gamma} \cos\theta_{k-1} \cos\theta_k + \beta(k) \sum_{\Gamma} \cos^2\theta_k \right]$$
$$= b^{-k} \beta(k) \sum_{\Gamma} \cos^2\theta_k = \frac{\beta(k)}{2b^k} \sum_{\Gamma} \left[1 + \cos(2\theta_k) \right] = \frac{\beta(k)}{2b^k} \left[b^k + \sum_{\Gamma} \cos(2\theta_k) \right] = \frac{\beta(k)}{2b^k} \left[b^k + \frac{b^k}{d}(2-d) \right]$$
$$= \frac{\beta(k)}{2} \left[1 + \frac{2-d}{d} \right] = \frac{\beta(k)}{d},$$

which determines the Lagrange multiplier

$$\beta(k) = dg(k). \tag{S99}$$

So the generalized probabilities are finally

$$P_{\Gamma} = \frac{1}{Z} \exp\left[\sum_{i=1}^{k} dg(i) \left(\vec{c}_{n_0} \cdot \vec{c}_{n_i} \right) \right], \tag{S100}$$

which is the probability for the whole trajectory. Due to the exponential form of this probability, we can decompose the trajectory probability into reorientation probabilities:

$$P_{\Gamma} = \prod_{i=1}^{k} P_{x,k}, \tag{S101}$$

given by:

$$P_{n_k,k} = \frac{1}{z} \exp\left[dg(k) \left(\vec{c}_{n_0} \cdot \vec{c}_{n_k} \right) \right], \tag{S102}$$

where z is the normalization constant for the reorientation probability.

Generalized time-correlated random walk: VACF decay analysis

Eq. (28) is at first sight, different from a simple power law decay. We now assess how similar Eq. (28) is to a simple power law decay for intermediate times. The easiest and most insightful way to achieve this is to expand both Eq. (28) and a generic power law in a Taylor series, and to compare the Taylor coefficients. We expand around $t = \Delta$. We will denote Eq. (28) by $C(t)$. The power law function has the following form:

$$G(t) = G_1 \left(\frac{\Delta}{t} \right)^{\gamma}, \tag{S103}$$

where the constants G_1 and γ are unspecified. First, we calculate the first two derivatives of $C(t)$:

$$\frac{\mathrm{d}C(t)}{\mathrm{d}t} = -\phi C_0 \Delta^{\phi} t^{-\phi-1} \operatorname{sech}^2\left[C_0\left(\frac{\Delta}{t}\right)^{\phi}\right] \tag{S104a}$$

$$\frac{\mathrm{d}^2C(t)}{\mathrm{d}t^2} = \phi C_0 \Delta^{\phi} t^{-2\phi-2} \operatorname{sech}^2\left[C_0\left(\frac{\Delta}{t}\right)^{\phi}\right]\left\{(\phi+1)t^{\phi} - 2\phi C_0 \Delta^{\phi} \tanh\left[C_0\left(\frac{\Delta}{t}\right)^{\phi}\right]\right\}, \tag{S104b}$$

with which we can calculate its Taylor series up to the second order term:

$$\begin{aligned} C(t) = {} & \tanh(C_0) - \frac{\phi C_0}{\Delta}\operatorname{sech}^2(C_0)(t-\Delta) + \\ & \frac{1}{2!}\frac{\phi C_0 \operatorname{sech}^2(C_0)}{\Delta^2}\{1+\phi[1-2C_0\tanh(C_0)]\}(t-\Delta)^2 + \mathscr{O}(t^3). \end{aligned} \tag{S105}$$

We now proceed in the same way with the power law:

$$\frac{\mathrm{d}G(t)}{\mathrm{d}t} = -G_1\Delta^{\gamma}\gamma t^{-\gamma-1} \tag{S106a}$$

$$\frac{\mathrm{d}^2G(t)}{\mathrm{d}t^2} = G_1\Delta^{\gamma}\gamma(1+\gamma)t^{-\gamma-2} \tag{S106b}$$

and expand in a Taylor series around $t = \Delta$:

$$G(t) = G_1 - \frac{G_1\gamma}{\Delta}(t-\Delta) + \frac{1}{2!}\frac{G_1\gamma}{\Delta^2}(1+\gamma)(t-\Delta)^2 + \mathscr{O}(t^3). \tag{S107}$$

To determine G_1 and γ we equate the zeroth and first order terms of Supplementary Eqs. (S105) and (S107), which yields

$$G_1 = \tanh(C_0) \tag{S108a}$$

$$\gamma = \phi C_0 \frac{\operatorname{sech}^2(C_0)}{\tanh(C_0)}, \tag{S108b}$$

so that the Taylor series expansion is determined by

$$\begin{aligned} G(t) = {} & \tanh(C_0) - \frac{\phi C_0}{\Delta}\operatorname{sech}^2(C_0)(t-\Delta) + \\ & \frac{1}{2!}\frac{\phi C_0 \operatorname{sech}^2(C_0)}{\Delta^2}\left[1+\phi C_0\frac{\operatorname{sech}^2(C_0)}{\tanh(C_0)}\right](t-\Delta)^2 + \mathscr{O}(t^3). \end{aligned} \tag{S109}$$

To estimate the similarity between both decays, we calculate the difference between Supplementary Eqs. (S105) and (S109) up to second order terms:

$$\begin{aligned} C(t) - G(t) \approx {} & \frac{1}{2!}\frac{\phi C_0 \operatorname{sech}^2(C_0)}{\Delta^2}\{1+\phi[1-2C_0\tanh(C_0)]\}(t-\Delta)^2 - \\ & \frac{1}{2!}\frac{\phi C_0 \operatorname{sech}^2(C_0)}{\Delta^2}\left[1+\phi C_0\frac{\operatorname{sech}^2(C_0)}{\tanh(C_0)}\right](t-\Delta)^2 = (t-\Delta)^2 \cdot \\ & \frac{1}{2!}\frac{\phi C_0 \operatorname{sech}^2(C_0)}{\Delta^2}\left\{1+\phi[1-2C_0\tanh(C_0)] - 1 - \phi C_0\frac{\operatorname{sech}^2(C_0)}{\tanh(C_0)}\right\} \\ & = (t-\Delta)^2\frac{1}{2!}\frac{\phi^2 C_0 \operatorname{sech}^2(C_0)}{\Delta^2}\left[1-2C_0\tanh(C_0) - C_0\frac{\operatorname{sech}^2(C_0)}{\tanh(C_0)}\right] \\ & = (t-\Delta)^2\frac{1}{2!}\frac{\phi^2 C_0 \operatorname{sech}^2(C_0)}{\Delta^2}\left\{1 - C_0\left[\frac{2\sinh(C_0)}{\cosh(C_0)} - \frac{1}{\cosh(C_0)\sinh(C_0)}\right]\right\} \\ & = (t-\Delta)^2\frac{1}{2!}\frac{\phi^2 C_0 \operatorname{sech}^2(C_0)}{\Delta^2}\left\{1 - C_0\left[\frac{2\sinh^2(C_0)+1}{\cosh(C_0)\sinh(C_0)}\right]\right\} \end{aligned}$$

which, after using hyperbolic identities, can be simplified to

$$C(t) - G(t) \approx (t-\Delta)^2\frac{1}{2!}\frac{\phi^2 C_0 \operatorname{sech}^2(C_0)}{\Delta^2}[1-2C_0\coth(2C_0)] \propto \left(\frac{\phi}{\Delta}\right)^2. \tag{S110}$$

LGCA simulations

Persistent random walk

Simulations were performed with only one particle with the reorientation probability given by Eq. (10) whose displacement and orientation were tracked at every time step. The lattice spacing was set to $\varepsilon = 0.25$, and the time step to $\tau = 0.015625$. The total simulation consisted of 100 time steps. The sensitivity (related to the internal force required for reorientation) was varied from $\beta = 3$ to $\beta = 5$. Simulations were repeated 1000 times for each sensitivity in order to obtain statistically relevant results. Simulation results for low and high sensitivities are shown in Fig. 5i.

As expected, correlations die off more slowly with increasing sensitivity. On the other hand, the MSD quickly starts behaving linearly, except for times close to zero, where it behaves almost ballistically. The region where displacement is almost ballistic increases with increasing sensitivity.

Additionally, we observe that the derived continuous time expressions agree perfectly with the discrete LGCA simulations.

Time-correlated random walk

Simulations were performed with only one particle with the reorientation probability given by Eq. (18). The lattice spacing was set to $\varepsilon = 0.25$, and the time step to $\tau = 0.015625$. The constant C_0 was set to 0.5, and the crossover time was equal to the time step length, $\Delta = \tau = 0.015625$. The total simulation consisted of 1000 time steps. Three different exponents were evaluated: $\phi = 0.1$, $\phi = 1$, and $\phi = 9$. Simulations were repeated 1000 times for each exponent, in order to obtain statistically relevant results. Simulation results for small and large exponents are shown in Fig. 5ii, as well as a plot of Eqs. (16) and (22) (integrated with MATLAB). We can see that Eqs. (16) and (22) match the simulation data perfectly. We also observe that for low values of the exponent ϕ the particle moves superdiffusively while for large values the particle diffuses normally.

Generalized time-correlated random walk

Simulations were performed with only one particle with probabilities given by Eq. (18). The lattice spacing was set to $\varepsilon = 0.25$ and the time step to $\tau = 0.015625$. The constant C_0 was set to 0.5 and the crossover time was equal to the time step length, $\Delta = \tau = 0.015625$. The total simulation consisted of 100 time steps. Two different exponents were evaluated, $\phi = 0.1$ and $\phi = 1$. Simulations were repeated 1000 times for each exponent, in order to obtain statistically relevant results. Simulation results for small, and large exponents are shown in Fig. 5iii as well as a plot of Eqs. (28) and (29) (integrated with MATLAB). We can see that Eq. (28) and (29) match the simulation data perfectly. Comparing Figs. 5ii and 5iii, it is evident that the VACF in both cases is quite similar, as expected given the small value of Δ used in these simulations. We also observe that for low values of the exponent ϕ the particle moves superdiffusively while for large values the particle diffuses normally.
